# Scalable Constant pH Molecular Dynamics in GROMACS

**DOI:** 10.1021/acs.jctc.2c00516

**Published:** 2022-09-21

**Authors:** Noora Aho, Pavel Buslaev, Anton Jansen, Paul Bauer, Gerrit Groenhof, Berk Hess

**Affiliations:** † Nanoscience Center and Department of Chemistry, University of Jyväskylä, 40014Jyväskylä, Finland; ‡ Department of Applied Physics and Swedish e-Science Research Center, Science for Life Laboratory, KTH Royal Institute of Technology, 100 44Stockholm, Sweden

## Abstract

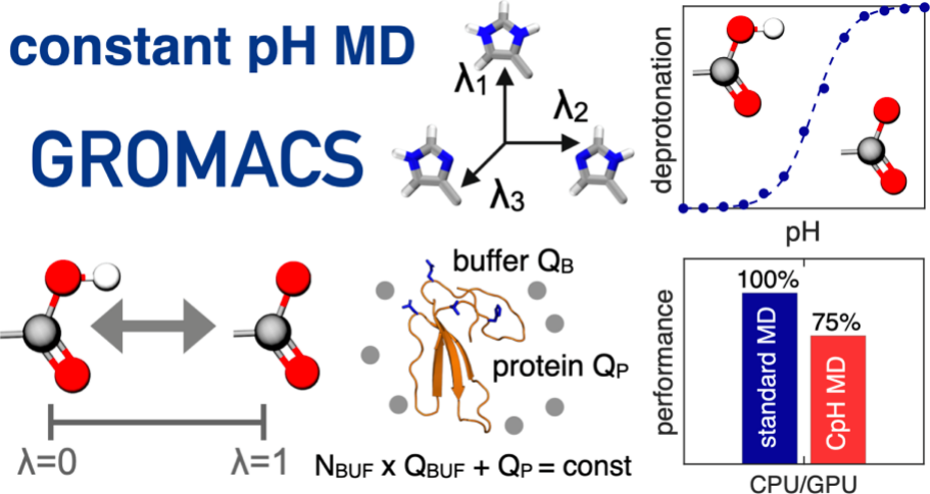

Molecular dynamics (MD) computer simulations are used routinely to compute atomistic trajectories of complex systems. Systems are simulated in various ensembles, depending on the experimental conditions one aims to mimic. While constant energy, temperature, volume, and pressure are rather straightforward to model, pH, which is an equally important parameter in experiments, is more difficult to account for in simulations. Although a constant pH algorithm based on the λ-dynamics approach by Brooks and co-workers [Kong, X.; Brooks III, C. L. *J. Chem. Phys.***1996**, *105*, 2414–2423] was implemented in a fork of the GROMACS molecular dynamics program, uptake has been rather limited, presumably due to the poor scaling of that code with respect to the number of titratable sites. To overcome this limitation, we implemented an alternative scheme for interpolating the Hamiltonians of the protonation states that makes the constant pH molecular dynamics simulations almost as fast as a normal MD simulation with GROMACS. In addition, we implemented a simpler scheme, called multisite representation, for modeling side chains with multiple titratable sites, such as imidazole rings. This scheme, which is based on constraining the sum of the λ-coordinates, not only reduces the complexity associated with parametrizing the intramolecular interactions between the sites but also is easily extendable to other molecules with multiple titratable sites. With the combination of a more efficient interpolation scheme and multisite representation of titratable groups, we anticipate a rapid uptake of constant pH molecular dynamics simulations within the GROMACS user community.

## Introduction

Since their introduction more than four decades ago, molecular dynamics (MD) computer simulations have come of age.^1^ Thanks to improvements in computer hardware, algorithmic developments, as well as increased accuracy of force fields, MD simulation has evolved into a predictive technique that can complement experiments by providing atomistic insights into the dynamics of complex systems.^1,2^ While many experimental conditions can be modeled with good accuracy, the aqueous proton concentration, or pH, is typically accounted for indirectly by constraining the protonation states of titratable residues to their, presumed, most probable form at the start of the simulation. Because the electrostatic interactions depend critically on the protonation state of the residues, the pH affects the conformational ensemble. Conversely, because the conformation can influence the proton affinity of the residues, or p*K*_a_, a direct correlation exists between pH and conformational dynamics, which cannot be captured if the protonation state is kept fixed in the simulation.^3^

To overcome this limitation in classical MD simulations and include the effect of pH on the conformational sampling directly, several solutions have been proposed in the last decades^4,5^ and used to investigate pH-dependent protein–protein^6^ and protein-RNA interactions,^7^ drug binding,^8,9^ and structural changes.^10,11^ These solutions can be roughly divided into a category that relies on discrete changes in protonation states^12−19^ and a second category in which a protonation state can change continuously.^20−33^ More recently, a third category that relies on the transfer of proton-like particles between titratable sites, including protein residues and solvent molecules, was proposed for the Martini force field.^34^

In the discrete constant pH approaches, the protonation state of a residue can change at regular intervals of the simulation according to a Metropolis Monte Carlo criterion.^16,17,28,35,36^ To avoid a low acceptance rate due to unfavorable solvent configurations, the Monte Carlo step is performed based on free energies calculated using the approximation of either an implicit solvent representation,^13,15^ or a short all-atom thermodynamic integration.^14,37^

Most continuous approaches for MD at constant pH are based on the λ-dynamics technique developed by Brooks and co-workers.^38^ A one-dimensional λ-coordinate with fictitious mass *m*_λ_ is introduced for each titratable site, and the equations of motion for these additional degrees of freedom are integrated along with the Cartesian positions of the atoms.^21^ The λ-coordinate defines the protonation state of the residue: at λ = 0 the residue is protonated and interacts with the rest of the system as such, while at λ = 1 it is deprotonated. The energy function that acts on the λ-coordinates depends on (i) the intrinsic proton affinity (reference p*K*_a_) of the titratable site in water, (ii) the interactions with the environment, which are mostly electrostatic,^39^ and (iii) the pH of the solvent, which is set by the user. In addition, potentials are introduced to bias sampling toward the physical states at λ = 0 and λ = 1. Protons are not transferred directly between the titratable residues and the solvent molecules but rather exchanged with an external proton bath. Because the chemical potential of this bath is determined by the proton concentration (pH) of the aqueous solution, constant pH MD (CpHMD) simulations based on λ-dynamics are performed in a grand canonical ensemble for the proton degrees of freedom.

While λ-dynamics-based constant pH approaches were originally developed for implicit solvent simulations,^21^ they have since then been adapted for explicit solvent simulations as well.^18,23−26,28,30^ The key computational challenge for explicit solvent implementations is the long-range electrostatic interaction, for which multiple solutions have been suggested, including a shifted cutoff scheme,^26^ a hybrid scheme combining the particle mesh Ewald (PME) treatment for the Cartesian coordinates with the generalized Born model for the λ-particles,^24,40^ and a fully consistent PME treatment for both λ and Cartesian degrees of freedom.^23,30^

In addition to accurate modeling of the long-range electrostatic interactions, also sampling can pose a serious challenge to simulations at constant pH. While the choice for the PME method in the original implementation of λ-dynamics in the fork of GROMACS 3.3 release^23^ was motivated by its accurate description of long-range electrostatics, the linear increase of the computational effort with the number of titratable sites limited the sampling efficiency, which meant that systems with many titratable sites could not be studied in practice.

To remove this bottleneck and enable constant pH MD with GROMACS at a modest additional cost compared to a standard simulation, we switch to an alternative scheme for computing the long-range electrostatic interactions of the λ-particles under periodic boundary conditions. The alternative scheme is based on a linear interpolation of partial charges^21^ rather than the potential energy functions as in the original implementation of constant pH MD in a GROMACS fork.^23^

Although the previous implementation of constant pH in a GROMACS fork was documented and shared with the community as an open-source program, there has been some misunderstanding about how electrostatic interactions were computed for λ-particles.^24,25,30^ To resolve this controversy, we first explain in detail how the electrostatic interactions were calculated in the previous GROMACS implementation of constant pH MD. We next contrast this linear interpolation between the potential energy functions of the protonated and deprotonated states of a residue on the one hand to the interpolation between the partial charges of both states on the other hand^21^ and show why the latter is computationally much more efficient. We then demonstrate the superior performance of the charge-interpolation scheme by running a series of constant pH MD simulations of amino acids and proteins. To emphasize that the new constant pH implementation in GROMACS is not restricted to a specific force field (or the resolution of a force field model) nor to a specific algorithm for evaluating electrostatic interactions, we also show the results of constant pH MD simulations with the Martini coarse-grained force field,^41^ in combination with a shifted cutoff electrostatics model. Because of GROMACS’ large user community, we expect our work to increase the popularity of constant pH MD simulations.

## Theory

Before discussing the differences between linear interpolating of the potential energy functions on the one hand,^23^ and of partial charges on the other hand,^21^ for computing the potential energy landscape of the titration coordinates, we briefly review the λ-dynamics approach^38^ that forms the basis for the constant pH molecular dynamics algorithm in GROMACS.^23^

### λ-Dynamics-Based Constant pH MD Simulations

A titratable site *i* can exist in a protonated or deprotonated state. The protonation state affects the interactions between the site and the rest of the system. In constant pH MD simulations based on λ-dynamics,^38^ an additional coordinate λ_*i*_ is introduced for each site *i*, and the potential energy function of the total system is continuously interpolated between the two protonation states along this coordinate, i.e., *V*(λ_*i*_) .^21^ A fictitious mass *m*_λ_ is assigned to each λ_*i*_-coordinate, and the coordinates evolve along with the Cartesian degrees of freedom of all atoms in the system, based on Newton’s equations of motion. Thus, the total Hamiltonian of the system is
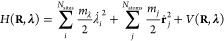
1where **R** is the vector of the Cartesian coordinates **r**_*j*_ of all *N*_atoms_ atoms with mass *m*_*j*_ and **λ** is the vector of the λ_*i*_ coordinates of all *N*_sites_ titratable sites.

### λ-Dependent Potential Energy Function

**Figure 1:**
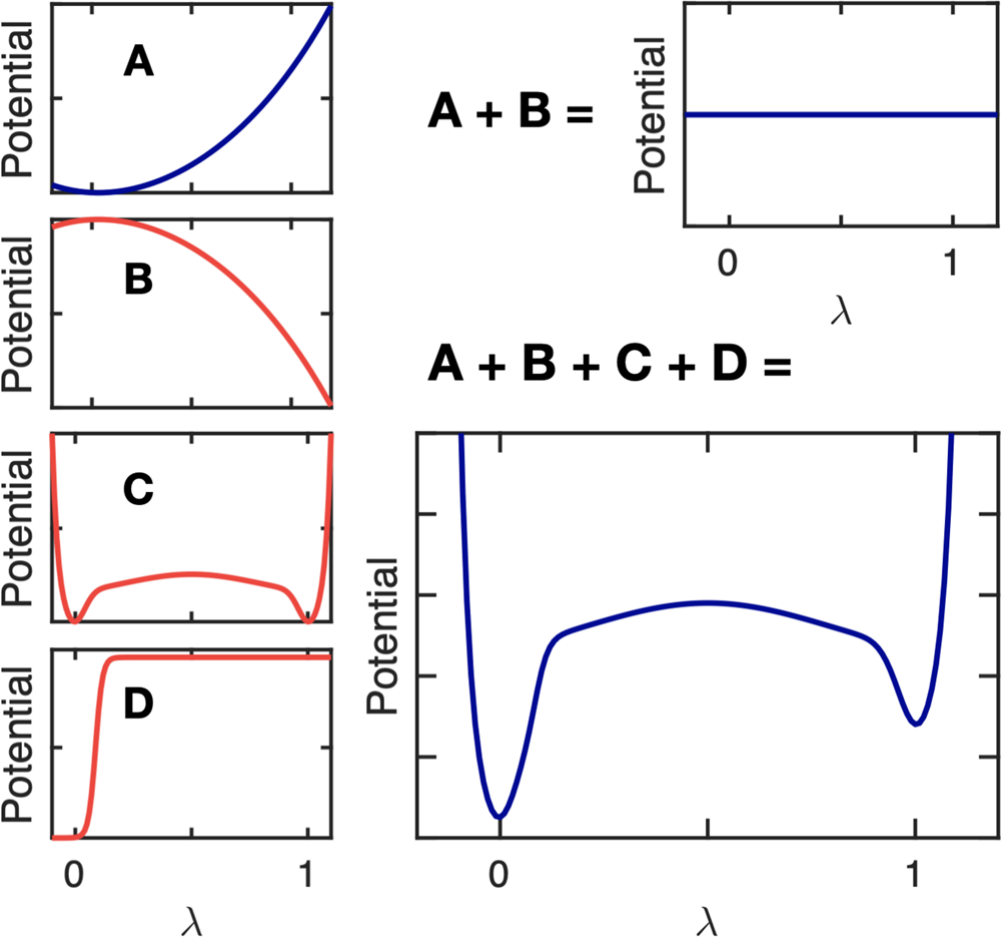
Illustration of the additional λ-dependent potential energy terms (B–D). Panel A shows the protonation free energy of a titratable residue in its reference state obtained at the force field level, Δ*G*_*i*_^MM^(λ_*i*_) . To compensate for the shortcomings of the force field and obtain a zero free energy difference between the two protonation states (A + B), we add the correction potential, *V*_*i*_^MM^(λ), shown in panel B. A biasing potential, *V*_*i*_^bias^(λ),^42^ is introduced to avoid sampling of nonphysical states (panel C). To model the proton chemical potential (pH), we add a pH-dependent term, *V*^pH^(λ_*i*_) (panel D). For a titratable residue at pH ≠ p*K*_a_, the total λ-dependent potential, including the interpolated force field functions and the three additional terms, is illustrated in panel (A + B + C + D).

In addition to the interpolation between the potentials of the protonated *V*_*A*_(**R**) and deprotonated states *V*_*B*_(**R**), three more λ-dependent terms are included in the potential energy function of the total system *V*(**R**, **λ**), as illustrated in Figure 1: (i) a correction potential *V*_*i*_^MM^(λ_*i*_) to compensate for missing quantum mechanical contributions to proton affinities (Figure 1B); (ii) a biasing potential *V*_*i*_^bias^(λ_*i*_) that enhances sampling of the physical end states at λ_*i*_ = 0 and λ_*i*_ = 1 (Figure 1C); and (iii) a pH-dependent term *V*^pH^(λ_*i*_) to model the chemical potential of protons in water (Figure 1D).

The purpose of adding the correction term *V*_*i*_^MM^(λ_*i*_) (Figure 1B) is to make the interpolated potential function flat if the titratable site *i* is in its reference state, for which the proton affinity is known experimentally, at pH = p*K*_a,*i*_. This potential is determined by evaluating the deprotonation free energy of the single residue in water (reference state) at the force field level by thermodynamic integration along the λ-coordinate (Figure 1A):
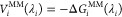
2

To prevent sampling of the nonphysical states between λ_*i*_ = 0 and λ_*i*_ = 1 on this flat potential energy surface while still enabling sufficient transitions between the physical end-states to sample both protonation states with the correct thermodynamic weight, we introduce the biasing potential *V*_*i*_^bias^(λ_*i*_) suggested by Donnini et al.^42^ (Figure 1C).

The pH-dependent term *V*^pH^(λ_*i*_) (Figure 1D) is a correction that includes the effect of the solution pH on the free energy difference between the protonated and deprotonated states, such that this difference is
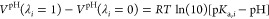
3where we use the experimentally determined p*K*_a,*i*_ value of residue *i* in its reference state. Although various forms for this potential have been suggested,^29,42,43^ we propose a smooth step-function-based potential:
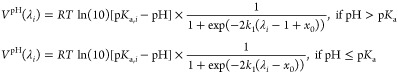
4where *k*_1_ and *x*_0_ define the steepness and kink position of the step function. In this form, illustrated in Figure 1D, the pH-dependent potential also aids in preventing the sampling of nonphysical states, i.e., 0.1 < λ < 0.9.

#### Linear Interpolation of Potential Energy Functions

In the previous implementation of constant pH MD in a GROMACS fork, the smooth interpolation of the force field potential energy function between the protonated and deprotonated states was achieved by linearly interpolating the force field potentials of these states.^23^ Thus, for a single titratable site, the λ-dependent potential is given by
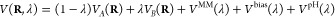
5with *V*^MM^(**R**, λ), *V*^bias^(λ), and *V*^pH^(λ) the correction, biasing, and pH-dependent potentials, respectively, that were briefly discussed above, and with short-hand notations for
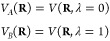
The gradient required for updating λ according to Newton’s equations of motion is
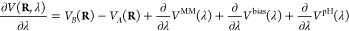
6Thus, the evaluation of the force on the λ-particle requires that the potential energy, including the long-range electrostatic interactions, is computed twice: once for λ = 0 (i.e., *V*_*A*_) and once more for λ = 1 (*i.e.*, *V*_*B*_). If the Particle-Mesh-Ewald (PME) method is used to compute those long-range electrostatic interactions,^44,45^ separate PME grid builds are needed because the charge distributions are not identical in states *A* and *B*.

For systems with many titratable sites, multiple λ-groups are introduced. Because the analytical expressions for the correction, biasing, and pH-dependent terms in eq 5 are additive, we no longer consider them explicitly in what follows and focus exclusively on the interpolation of the force field potential energies between the multiple protonation states of the system. For *N λ*-coordinates, there are 2^*N*^ such states and the interpolation generalizes to^20^
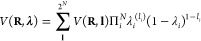
7Here, we represent the *N λ*_*i*_-coordinates as an *N*-dimensional vector **λ**. The 2^*N*^ possible protonation states of the system are represented by the *N*-dimensional vector **l** with elements *l*_*i*_ equal to 0 or 1 that indicate whether a site *i* is protonated (λ_*i*_ = 0) or deprotonated (λ_*i*_ = 1). The sum runs over all 2^*N*^ possible combinations of *l*_*i*_ = 0 and *l*_*i*_ = 1. The gradient required for updating λ_*i*_ is obtained by deriving the interpolated potential, *V*(**R**, **λ**), with respect to λ_*i*_:
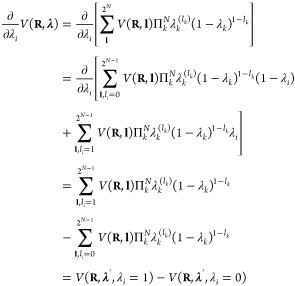
8where the ′ indicates that λ_*i*_ is omitted from vector **λ**. Note that, as we focus only on the interpolated potentials, the biasing, correction, and pH-dependent terms are left out.

In general, the number of terms in the potential (eq 7) increases exponentially with the number of titratable sites. However, for pairwise interactions involving titratable sites whose nonbonded force field parameters do not depend on the protonation state of the other sites (chemically uncoupled sites), the number of terms required to evaluate the interpolated potential scales linearly. For systems with such “chemically”, or “topologically” uncoupled sites, the interpolated potential contains four types of interactions
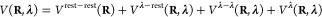
9For pairwise electrostatic interactions, the terms on the right-hand side are defined as

1. Interactions between atoms that are not part of any λ-group, and hence independent of the λ_*i*_’s:
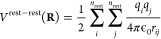
10where the sums run over all *n*_rest_ atoms that are not part of a λ-group.
2. Interpolated interactions between atoms of each λ-group with atoms that are not part of any λ-group:
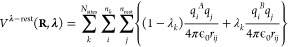
11where the first sum runs over all titratable sites, the second one runs over all *n*_*k*_ atoms of the *k* th λ-group, and the final sum runs over all atoms that are not part of any λ group.
3. Interpolated interactions between atoms belonging to two different λ-groups:
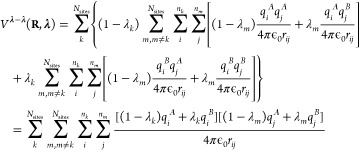
12
4. Interpolated interactions within each of the λ-groups:
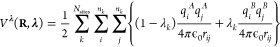
13

From eq 8, the gradient with respect to λ_*k*_ is
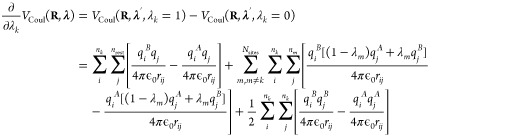
14Thus, the evaluation of the Coulomb contribution to the gradient for each λ_*k*_-group requires two electrostatic computations, with the interpolated partial charges of the other λ_*m*_ sites (i.e., *q*_*j*_(λ_*m*_) = (1 – λ_*m*_)*q*_*j*_^*A*^ + λ_*m*_*q*_*j*_^*B*^):
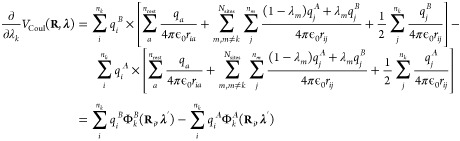
15Here, we introduced the electrostatic potential Φ_*k*_^*A*^(**R**_*i*_, **λ**′) of the system with partial charges of λ-group *k* in the protonated state (*q*_*i*_^*A*^) and interpolated charges for all other λ-groups. As before, **λ**′ is the vector with all λ_*m*_’s except λ_*k*_. Likewise, electrostatic potential Φ_*k*_^*B*^(**R**_*i*_, **λ**′) is evaluated with the partial charges of λ-group *k* in the deprotonated state (*q*_*i*_^*B*^) and the same interpolated charges for all other λ-groups. Thus, 2*N*_sites_ computations are needed to evaluate the gradients for all titratable sites. The same arguments apply to pairwise Lennard-Jones interactions, but because the contribution of Lennard-Jones interaction to p*K*_a_ shift is minor, we neglected them in this work (see Supporting Information).

#### Linear Interpolation of Partial Charges

While the linear scaling of the gradients for the pairwise potentials in eq 15 in principle is a great improvement over the formal exponential scaling in eq 8, the requirement of performing 2*N*_sites_ calculations per MD step still poses a computational bottleneck, in particular for larger systems. To overcome this bottleneck for electrostatic interactions, we follow the suggestion by Brooks and co-workers to interpolate charges rather than interaction functions.^21^ When interpolating the partial charges between the protonation states of *N*_sites_ chemically uncoupled titratable sites, the λ-dependent Coulomb energy becomes
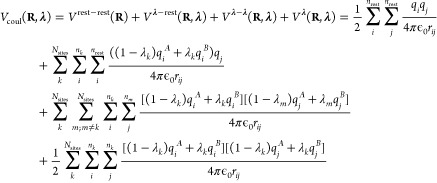
16The gradient of the potential energy with respect to λ_*k*_ is
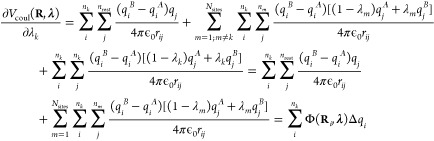
17where Φ(**R**_*i*_, **λ**) is the electrostatic potential at the position of atom *i* due to the charge distribution of all other atoms in the system, including the atoms of all titratable sites, for which the partial charges are interpolated:
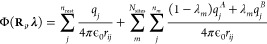
and Δ*q*_*i*_ is the difference between the atomic charges of titratable residue *i* in the protonated (A) and deprotonated (B) states:
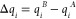
In contrast to when potential energy functions are interpolated, the same electrostatic potential is used to evaluate the electrostatic forces on both the atoms and the λ-particles. Therefore, a single electrostatic calculation per time step suffices. If the electrostatic interactions are modeled with the smooth Particle Mesh Ewald method,^44,45^ the short-range real-space interactions and long-range reciprocal-space interactions are computed separately. For the pairwise short-range interactions in real space, an additional calculation for each interacting pair and a subsequent accumulation of the potential at each atom is needed. Whereas this calculation comes at no extra computational cost if the standard pair interaction kernels are used, the accumulation leads to a measurable computational overhead, as we will show later. For the mesh part of the PME calculation, a gathering of potentials from the grid is required for charges in λ-groups only, but this also comes at a negligible computational overhead. Because the extra effort required to compute the gradients on the λ-particles is rather small, a constant pH MD implementation based on charge interpolation is computationally not much more expensive than a normal MD simulation, which is a major improvement with respect to the previous CpHMD implementation in GROMACS.^23^

### Multisite Representation of Chemically Coupled Titratable Sites

**Figure 2:**
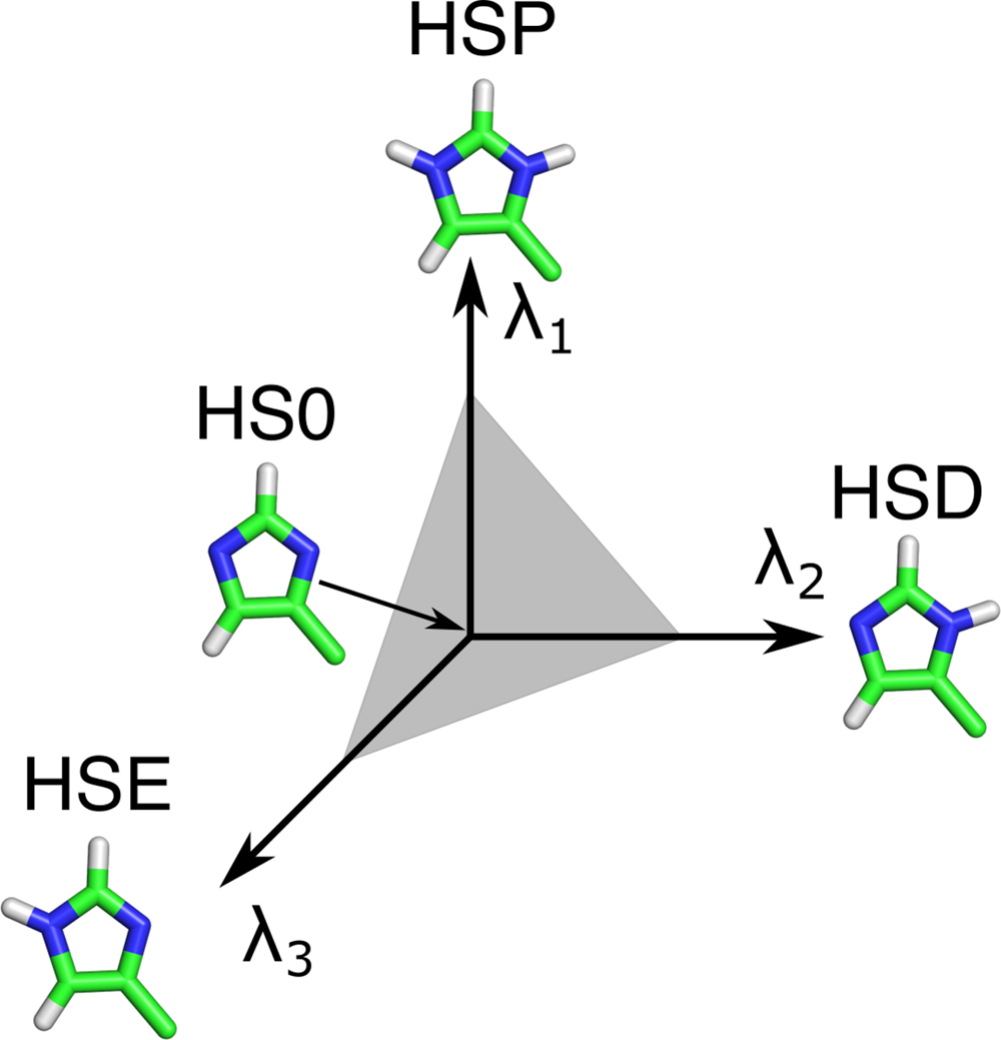
Multisite representation illustrated for a histidine side chain. Each possible protonation state is represented by its own λ-coordinate. HSP refers to doubly protonated histidine, HSD, and HSE to histidine singly protonated at the *N*_δ_ or *N*_ϵ_, respectively. HS0 is a common, nonphysical state of the residue. To restrict the sampling to the plane connecting the physical states, a constraint λ_1_ + λ_2_ + λ_3_ = 1 is applied (gray plane). A biasing potential is also applied to enhance sampling at the end states, where one of the λ-coordinates is close to one, while the other coordinates are close to zero.

If titratable sites are “chemically” or “topologically” coupled, the force field parameters of one site depend on the value of the λ-coordinate of the other site, and *vice versa*. For example, histidine can exist in three protonation states, as shown in Figure 2. In most force fields, the partial charges of all atoms in the His side chain, including the two sites, depend on the protonation state. Hence, if the N_δ_ site changes protonation, the electrostatic interactions of the N_ϵ_ site are also affected.

To model the chemically coupled sites in the histidine side chain, Khandogin and Brooks introduced two λ-coordinates:^22^ one that interpolates between the double and single protonated forms and a second coordinate switching between protonation at the N_δ_ and the N_ϵ_ atoms. Donnini et al. introduced separate λ-coordinates for N_δ_ and N_ϵ_.^23^ In both solutions, the coupling between the coordinates is achieved with a two-dimensional correction potential.

Because extending the dimensionality beyond two coordinates is difficult from both the implementation and parametrization perspective, Brooks and co-workers introduced a multisite representation,^46,47^ where a separate λ_*i,k*_-coordinate is assigned to each physical state *k* of a titratable group *i*. For a residue with multiple “chemically”-coupled titratable sites, each λ_*i,k*_-coordinate has the same state at λ_*i*,*k*_ = 0, while at λ_*i*,*k*_ = 1, the group is in one of the *n*_*i*_ possible protonation states (i.e., state *k*) of residue *i*. The state at λ_*i*,*k*_ = 0 is the same for all λ_*i,k*_-coordinates in residue *i* but does not correspond to a physical protonation state of the residue and neither do states for which the sum of the λ_*i,k*_-s is not equal to 1 (Figure 2). To restrict sampling to the (hyper-)plane connecting the physical states, the sum of the λ_*i,k*_ values is constrained (∑_*k*_λ_*i,k*_ = 1). Since the λ-dynamics implementation in a fork of GROMACS relies on linear λ-coordinates, rather than on auxiliary circular coordinates that would fulfill the constraints by construction,^46,48^ we apply a constraint on the sum of λ_*i,k*_-coordinates. To efficiently apply this constraint, we use an analytical expression to solve a generalized version of the charge constraint introduced by Donnini et al.^42^ (see the Appendix). While an atom can be part of multiple λ_*i,k*_-coordinates in residue *i*, each affecting its charge, we show in the Supporting Information that the expression for the contribution of this atom to the total Coulomb energy is identical to that of an uncoupled site (eq 17).

In the multisite representation, each λ-coordinate is independent of the others and thus evolves on a one-dimensional potential (eq 4), similar to that of “chemically” uncoupled sites. However, in contrast to the uncoupled sites, the correction potential *V*^MM^ is multidimensional as its value depends on all λ_*i,k*_-coordinates representing each of the possible protonation states of residue *i*. These potentials are obtained through a least-squares fit of a multidimensional polynomial to the ensemble-averaged gradients of the potentials with respect to λ_*i,k*_ evaluated on the (*n*_*i*_ – 1)-dimensional grid of the *n*_*i*_ coupled λ-coordinates, *i.e.*, ⟨∂ *V*/∂λ_*i,k*_⟩_λ_1_...λ_*ni*__. The fitting procedure is explained in detail in the Supporting Information.

The multisite representation can be applied to residues with any number of titratable sites, including residues with only a single titratable site. In the latter case, two λ-coordinates, corresponding to the protonated state (λ_*i*,1_ = 1, λ_*i*,2_ = 0) and deprotonated state (λ_*i*,1_ = 0, λ_*i*,2_ = 1), are introduced with a constraint on their sum (λ_*i*,1_ + λ_*i*,2_ = 1).

## Methods

We have implemented the algorithms for CpHMD with charge interpolation in a fork of GROMACS software package (2021 release).^49^ The code and manuals are available for free at https://gitlab.com/gromacs-constantph/constantph. Here we verify the validity of our implementation for reproducing p*K*_a_ values of peptides and proteins. To demonstrate that the linear interpolation of charges (eq 17) scales better with the number of titratable sites in the system than the linear interpolation of interaction functions (eq 15), we compared the scaling between our new implementation, which is based on linear charge interpolation on the one hand, and a previous implementation in a fork of the GROMACS 3.3 release, which is based on linear interpolation of the force field potentials on the other hand.^23^ To estimate the additional computational effort required for performing CpHMD with the new implementation, we also compared the performance of a CpHMD simulation to that of a normal MD simulations on both CPUs and GPUs.

### Simulated Systems

To test the implementation, we performed constant pH MD simulations of the following systems: (1) glutamic acid (Glu), (2) aspartic acid (Asp), (3) histidine (His), (4) Cardiotoxin V (PDB ID: 1CVO(50)), (5) hen egg white lyzozyme (HEWL, PDB ID: 2LZT(51)), (6) the GLIC pentameric ligand-gated ion channel (PDB ID: 4HFI(52)), and (7) turkey ovomucoid inhibitor (PDB ID: 2GKR(53)).

In systems 1–6, the interactions were modeled with the CHARMM36^54^ all-atom (AA) force field, with some modifications in the torsion parameters to accelerate the convergence. These modifications are presented and validated in an accompanying paper, in which we report the application of our constant pH implementation to lysine, C-, and N-termini.^55^ Systems 1–5 were also simulated with the Martini 2.0 coarse grained (CG) force field.^41^ The martinize.py script was used to automatically generate the CG representation of these systems.^56^ System 7 was simulated to compare the efficiency of interpolating charges and potentials. The interactions in this system were modeled with the OPLS force field^57^ because the GROMACS 3.3 release, on which the linear interpolation of potentials implementation was based, does not support the CMAP correction that is needed for the CHARMM36 force field.^58^

The amino acids Glu, Asp, and His were modeled as tripeptides Ala-X-Ala with acetylated N-terminus (ACE) and *N*-methylamidated C-terminus (CT_3_). The proteins were simulated with charged termini. The tripeptides were placed in a periodic rectangular box of dimensions 5 × 5 × 5 nm^3^ with approximately 4000 CHARMM TIP3P^59,60^ water molecules in the AA simulations and 950 polarizable water particles in the CG simulations.^61^ The water-soluble protein cardiotoxin V was placed in a periodic rectangular box of 7.9 × 7.9 × 7.9 nm^3^ and filled with 16500 CHARMM TIP3P water molecules in the AA simulations. In the CG simulations, the protein was placed inside a periodic rectangular box of 5.7 × 5.7 × 5.7 nm^3^ and filled with 1800 polarizable water particles. The larger water-soluble protein HEWL was placed in a periodic rectangular box of 8.9 × 8.9 × 8.9 nm^3^ and filled with 23000 CHARMM TIP3P water molecules in the AA simulations and 5400 polarizable water particles in the CG simulations. Na^+^ and Cl^–^ ions were added to all systems at 0.15 M concentration to neutralize the protein systems. The turkey ovomucoid inhibitor protein was placed in a box of 4.9 × 4.9 × 4.9 nm^3^ with 3086 SPC^62^ water molecules. The GLIC protein was embedded into a bilayer membrane containing 498 phosphatidylcholine (POPC) lipids, placed in a box of 14.0 × 14.0 × 15.9 nm^3^, and filled with 66494 CHARMM TIP3P waters, 58 Na^+^, and 123 Cl^–^ ions. The system contained 292135 atoms in total. The simulation of this system was performed with the GROMACS 2021.4 release as reference. The GLIC benchmarks were run with default settings on an Intel i9–7920X 12-core CPU and an Nvidia RTX 2080 Ti GPU. All input configurations are provided as the Supporting Information.

In the AA simulations, Coulomb interactions were modeled with the smooth PME method with a real-space cutoff of 1.2 nm and a grid spacing of 0.14 nm,^44,45^ while Lennard-Jones interactions were smoothly switched to zero in a range from 1.0 to 1.2 nm. In the CG simulations, Coulomb interactions were modeled by a Reaction Field potential with a 1.1 nm cutoff, ϵ_r_ = 2.5, and ϵ_RF_ = ∞, while Lennard-Jones interactions were truncated at 1.1 nm.^63^ To keep the temperature constant at 300 K, we used the v-rescale thermostat^64^ with time constants of 0.5 and 1.0 ps^–1^ for AA and CG simulations, respectively. The pressure was kept constant at 1 bar with the Parrinello–Rahman barostat^65^ with relaxation times of 2.0 and 12.0 ps for AA and CG simulations, respectively. A leapfrog integrator was used with an integration step of 2 and 20 fs for AA and CG simulations, respectively. In the AA simulations, the LINCS algorithm^66^ was used to constrain h-bond lengths of the solutes, while the SETTLE^67^ algorithm was used to constrain internal degrees of freedom of the water molecules. Prior to the constant pH MD simulations, the potential energy of each system was minimized using the steepest descent method, followed by 1 ns of equilibration.

### Constant pH MD Simulation Setups

In the atomistic simulations, the multisite representation was used to model the protonation states of titratable residues. Two λ-coordinates were introduced to model the two forms of the carboxylic acid side chain in Asp and Glu, while three coordinates were used to describe the three protonation states of the imidazole side chain in His. In the CG simulations, the single-site representation was used, in which the A and B states represent the protonated and deprotonated states of the titratable beads. Because, in contrast to AA force fields, there is no distinction between the two neutral forms of the His side chain in the Martini force field, the single-site description for HIS suffices in the CG simulations. In both atomistic and coarse-grained simulations, the transformations between the different protonation states were achieved by changing the charges of the ionizable groups. The Lennard-Jones and bonded terms (bonds, angles, and torsions) were kept in the protonated and deprotonated states in AA and CG simulations, respectively. We show in Figure S3 that the contribution of these terms is sufficiently small to be neglected without significant error. We note, however, that these terms can be made λ-dependent as well, but this is beyond the scope of the current work since the efforts to implement this are high.

The mass of the λ-particles was set to 5 atomic units, and their temperature was maintained at 300 K by using a separate v-rescale thermostat for the λ-coordinates with a time constant of 2.0 ps^–1^. For all λ-coordinates the biasing potential *V*_*i*_^bias^(λ_*i*_) was defined by equation S1 in the Supporting Information. The barrier height of the double-well potential was set to 5.0 and 7.5 kJ/mol for AA and CG simulations, respectively. The parameters for the double-well potential and the pH-dependent potential (eq 4) are provided in Table S1.

For the tripeptides, we calculated five independent CpHMD trajectories of 20 ns each at 13 pH values, ranging from 1.0 to 7.0 for the peptides with Glu and Asp, and from 4.0 to 10.0 for the peptides with His. For the cardiotoxin V protein (three Asp and one His titratable residues), we performed five independent CpHMD simulations of 50 ns at 15 pH values between 1.0 to 8.0. For the HEWL protein (seven Asp, two Glu, and one His titratable residues), we performed five independent CpHMD simulations of 75 ns at 21 pH values between −1.0 to 9.0. The values of the λ-coordinates were written to the output file with a frequency of 1 ps^–1^.

#### Reference States and Force Field Correction Potentials

The constant pH simulations of the aforementioned systems require reference states for Asp, Glu, and His, in which the proton affinity (p*K*_a_) is known from the experiment. The measured and calculated (force field) deprotonation free energies of these reference states were used to include the effect of the pH bath, *V*^pH^(λ), as well as the effects of the breaking and forming of chemical bonds in the simulation, i.e., *V*^MM^ in eq 2. The measured reference p*K*_a_ values used in this work are included in Table 1. Note that the experimental values were obtained for pentapeptides, while tripeptides were used for computing *V*^MM^. This however did not affect the results, as shown in Figure S2.

**Table 1:** p*K*_a_ Values Obtained from Titration Simulations^a^

	tripeptide simulations^69,70^		
	p*K*_a_ values		
amino acid	CHARMM36	MARTINI	exp.
Asp	3.61 ± 0.03	3.69 ± 0.02	3.65
Glu	4.26 ± 0.04	4.30 ± 0.03	4.25
His macroscopic	6.34 ± 0.08	6.40 ± 0.03	6.42
His HSD	6.56 ± 0.06		6.53
His HSE	6.90 ± 0.05		6.94

	simulation of cardiotoxin V^71,72^		
	p*K*_a_ values		
amino acid	CHARMM36	MARTINI	exp.
His-4	5.14 ± 0.16	4.54 ± 0.09	5.5
Glu-17	4.08 ± 0.08	4.36 ± 0.04	4
Asp-42	4.02 ± 0.10	4.30 ± 0.05	3.2
Asp-59	2.41 ± 0.07	1.45 ± 0.03	<2
	*r* = 0.96	*r* = 0.80	
	MSE = 0.24	MSE = 0.64	
	RMSE = 0.49	RMSE = 0.80	

	simulation of HEWL^73^		
	p*K*_a_ values		
amino acid	CHARMM36	MARTINI	exp.
Glu-7	2.82 ± 0.07	4.86 ± 0.05	2.6
His-15	4.84 ± 0.05	5.42 ± 0.05	5.5
Asp-18	3.35 ± 0.05	3.31 ± 0.03	2.8
Glu-35	7.64 ± 0.13	6.36 ± 0.05	6.1
Asp-48	0.99 ± 0.07	3.36 ± 0.05	1.4
Asp-52	5.69 ± 0.12	7.18 ± 0.10	3.6
Asp-66	1.70 ± 0.10	5.22 ± 0.05	1.2
Asp-87	1.73 ± 0.03	3.47 ± 0.05	2.2
Asp-101	5.43 ± 0.11	4.20 ± 0.06	4.5
Asp-119	2.77 ± 0.05	3.80 ± 0.05	3.5
	*r* = 0.90	*r* = 0.49	
	MSE = 0.96	MSE = 4.01	
	RMSE = 0.98	RMSE = 2.00	

a The reference p*K*_a_ values for tripeptides are given in the last column that contain the experimental p*K*_a_ values. The values for Asp and Glu are taken from ref (69), while the microscopic and macroscopic p*K*_a_ values for His are taken from ref (70). Experimental p*K*_a_ values for cardiotoxin V are from refs (71 and 72) and for HEWL from ref (73). For both proteins Pearson correlation (*r*), MSE and RMSE errors are provided.

Thermodynamic integration was used to compute the reference free energies as follows: the partial charges in tripeptide systems representing the reference states of Glu, Asp, and His were linearly interpolated between λ = −0.1 and λ = 1.1 with a step of 0.05 under the constraint λ_1_ + λ_2_ = 1 for Glu and Asp, while for His, the constraint was λ_1_ + λ_2_ + λ_3_ = 1. For each set of λ values, called a grid point, a 10 ns MD simulation was performed. The ∂*V*/∂λ_*i*_ values were saved every ps, which is approximately equal to the autocorrelation times for the λ-coordinates. The total charge of the system was kept neutral by simultaneously changing the charge of a single buffer particle, as discussed below. The ∂*V*/∂λ_*i*_ values were averaged over the last 9 ns of the trajectories. To obtain an analytical expression for *V*^MM^, a fifth-order polynomial was fitted to these averages for Asp and Glu, while an eighth-order polynomial was fitted for His, taking into account possible linear dependencies of the coefficients (see the Supporting Information). Fitting errors were below 0.5 kJ/mol for Asp and Glu and below 1 kJ/mol for His, which are of similar magnitude as the statistical accuracy of the derivatives.

#### Buffer Particles

Dynamically changing partial charges can affect the total charge of the simulation unit cell, which can lead to artifacts, as documented for instance in Hub et al. for Ewald-based methods.^68^ To avoid such artifacts, it is essential to keep the total charge of the unit cell constant. Two approaches have been proposed: (i) direct coupling between each titratable residue and a water,^27^ or ion,^25^ and (ii) titratable buffers that collectively compensate for changes in charge of all titratable residues.^42^

Here, we follow the latter approach, but with several improvements for all-atom simulations. First, to avoid restraints, which were needed to minimize interactions between the buffers and the titrable sites in previous work,^42^ we introduced buffer particles with both small LJ radius and small partial charges of maximal |0.5|*e*, such that they do not disturb the hydrogen bond network, nor interact too strongly with the titratable sites or other buffers. Second, to also prevent strong interactions with hydrophobic regions in the system, the C^(6)^ dispersion parameter with anything other than water was set to zero, including the other buffers. The latter also avoids the clustering of buffers during the simulation. Thus, the buffer particles have an σ of 0.25 nm and an ϵ of 4 kJ/mol. Further details on buffer parametrization are provided in the accompanying paper.^55^ In coarse-grained simulations, standard Na^+^ ions were used as buffer particles.

As in Donnini et al.,^42^ the buffers were collectively coupled to the titratable sites in the system via a charge constraint. The charges of all buffers were thus simultaneously interpolated between −0.5*e* and 0.5*e*, keeping the simulation box neutral. For all peptide simulations, 10 such buffers were introduced into the system, while 20 and 50 buffers were added to the simulation boxes with cardiotoxin V and HEWL proteins (systems 4–5), respectively, in both AA and CG models. 185 buffer particles were used in GLIC simulations.

### Analysis of the Constant pH Trajectories

To estimate the p*K*_a_ values of titratable groups from multiple simulations at various pH values, we computed the average fraction of deprotonated frames (*S*^deprot^) over all replicas. For a group with a single titratable site, this average was obtained as
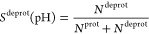
18where *N*^prot^ and *N*^deprot^ are the total number of frames in which the site is protonated and deprotonated, respectively. For titratable sites modeled in the single-site representation, we considered it protonated if λ is below 0.2 and deprotonated if λ is above 0.8. For sites that are described with the multisite description, we considered a state protonated if the λ associated with the protonated form of the residue is above 0.8 and deprotonated if the λ associated with the deprotonated form of the residue is above 0.8.

To estimate the macroscopic p*K*_a_ values of histidine, which contains two titratable sites N_ϵ_ and N_δ_, we calculated for each pH value the average fraction of frames in which the residue is deprotonated at either of the two sites:
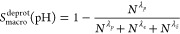
19where *N*^λ_*p*_^, *N*^λ_ϵ_^, and *N*^λ_δ_^ are the numbers of frames in which λ_*p*_ > 0.8, λ_ϵ_ > 0.8, and λ_δ_ > 0.8 (Figure 2). To estimate the microscopic p*K*_a_ values for the two sites of His, we calculated for each site the average fraction of frames in which that site was deprotonated:
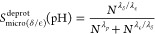
20Errors were estimated from the standard error of the mean for the different replicas.

The averaged fractions at each pH value were fitted to the Henderson–Hasselbalch equation:
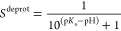
21which yielded the p*K*_a_ values as fitting parameters. The error in the p*K*_a_ was estimated from the 95% confidence interval for the nonlinear least-squares fit to the average (*S*^deprot^) values.

## Results and Discussion

Here we discuss the results obtained with our new implementation of constant pH into the fork of the GROMACS 2021 release.^49^ While here our focus is on the validity and performance of the constant pH MD implementation, the convergence of the conformational and λ degrees of freedom are investigated systematically in the accompanying paper.^55^

### Titration of Single Amino Acids

**Figure 3:**
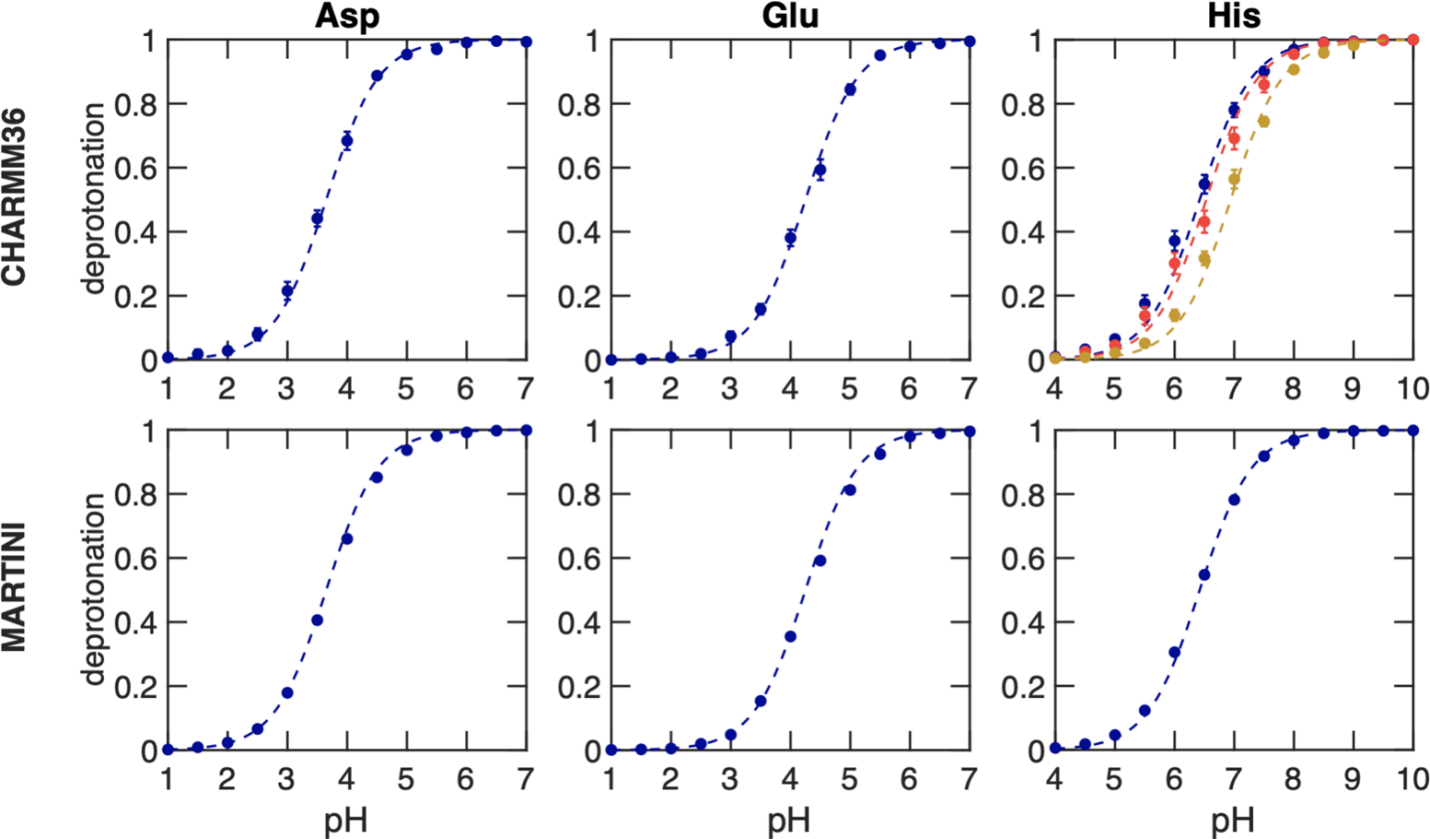
Titrations of tripeptide amino acids (Glu, Asp, and His) in water. The top and bottom rows show titrations performed with the modified AA CHARMM36^55^ and CG Martini^41^ force fields, respectively. In all simulations, neutrality was maintained by including ten buffer particles in combination with the charge constraint. Dots show the fraction of frames in which the residue is deprotonated, and the dashed lines represent the fits to the Henderson–Hasselbalch equation. For His, the blue color represents the macroscopic p*K*_a_, while yellow and red represent the microscopic p*K*_a_ values for HSD (proton on N_δ_) and HSE (proton on N_ϵ_), respectively. In the Martini 2.0 model, HSD and HSE are indistinguishable and hence only the macroscopic titration curve is shown. Errors of *S*^deprot^ were estimated from the standard error of the mean for the different replicas. From the fits, the p*K*_a_ values were estimated and listed in Table 1.

In Figure 3, we show the titration curves for AlaAspAla, AlaGluAla, and AlaHisAla tripeptides, obtained from simulations with the modified all-atom CHARMM36^55^ and coarse-grained Martini 2.0 force fields.^41^ Fitting the deprotonated fractions as a function of pH value to the Henderson–Hasselbalch equation (dashed lines in Figure 3) yields p*K*_a_ values for the tripeptides that are within 0.1 p*K*_a_ units from the reference values. Comparing the titration curves obtained with the Martini 2.0 force field in our implementation to those computed with the constant pH approach developed explicitly for this coarse-grained model,^34^ our results suggest a much better agreement with the experiment than the latter. We attribute this difference to the more sophisticated explicit treatment of proton-like particles in the Martini constant pH approach. The rather good agreement between the titration curves obtained for both force fields on the one hand and the experiment on the other hand suggests that our implementation has little to no dependency on the force field, in line with the GROMACS philosophy of supporting a wide range of popular force fields.

### Titration of Proteins

**Figure 4:**
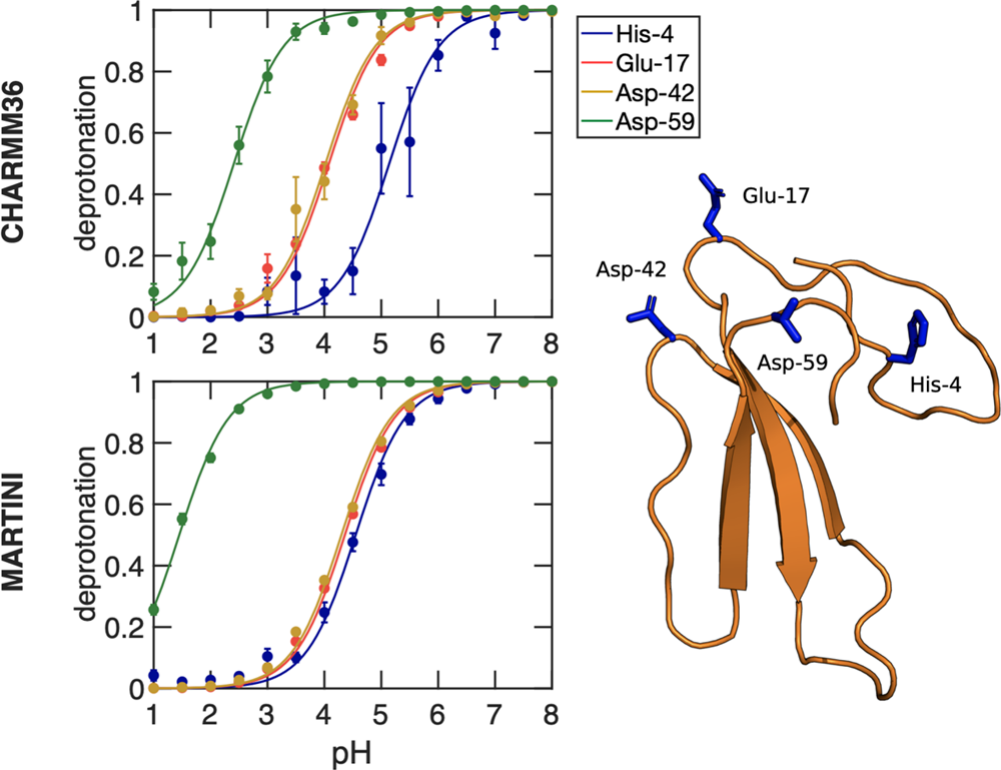
Titration curves of the cardiotoxin V protein obtained from constant pH MD simulations with the CHARMM36 (top) and Martini 2.0 force fields (bottom). For each of the four titratable residues in this protein, the dots show the fraction of frames in which the residue is deprotonated. Errors of *S*^deprot^ were estimated from the standard error of the mean for the different replicas. The lines show the best fits to the Henderson–Hasselbalch equation. The p*K*_a_ values for each titratable residue were estimated from these fits and listed in Table 1. The right panel shows the protein structure with the four titratable residues highlighted in stick representation.

**Figure 5:**
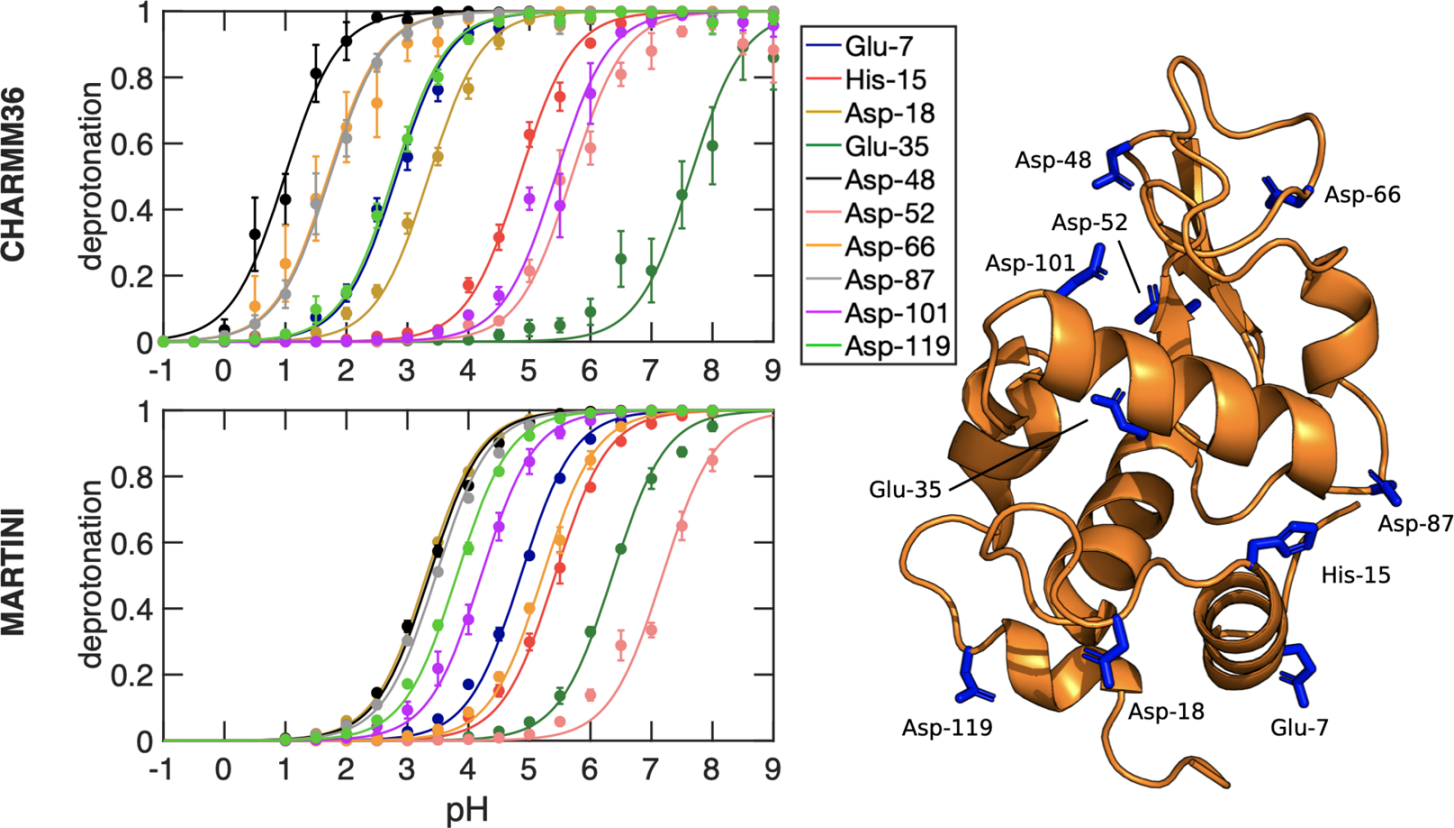
Titration curves of the HEWL protein obtained from constant pH MD simulations with the CHARMM36 (top) and Martini 2.0 force fields (bottom). For each of the ten titratable residues, the dots show the fraction of frames in which that residue is deprotonated. Errors of *S*^deprot^ were estimated from the standard error of the mean for the different replicas. The lines show the best fit to the Henderson-Hasselbach equation. The p*K*_a_ values for each titratable residue were estimated from these fits and listed in Table 1. The right panel shows the protein structure with the ten titratable residues highlighted in stick representation.

The titration curves of cardiotoxin V and HEWL proteins are shown in Figures 4 and 5, respectively. The p*K*_a_ values obtained from fitting the Henderson–Hasselbalch equation to the degree of deprotonation in the all-atom simulations of both proteins with the CHARMM36 force field, listed in Table 1, are in good agreement with previous constant pH MD simulations^30,74^ and in reasonable agreement with experimental estimates from NMR spectroscopy [Pearson correlation coefficient (*r*) 0.96 and 0.9, RMSE 0.49 and 0.98 for cardiotoxin V and HEWL, respectively].^71−73^ The p*K*_a_ values and *S*^deprot^ are converged in 50 ns, as discussed in the Supporting Information section 5 (Figures S6–S9). Analysis of the RMS deviation of the backbone and of the RMS fluctuation of the residues, plotted in Figures S10–S13, suggest no major influence of the pH on the structural stability of these proteins. The titration correlates with solvent exposure, which contributes to the stabilization of the charged protonation state (see Supporting Information section 7) (Figures S14–S16). The trends in the p*K*_a_ shifts are well reproduced, including the downshift of Asp-59 in cardiotoxin V, and, with the exception of Glu-35 and Asp-52 in HEWL, the deviations are below 1 p*K*_a_ unit. We note that also in previous constant pH simulations with the CHARMM force field,^22,30^ similar deviations were found for these two residues (see Figure S5). This suggests that the origin of the discrepancy might lie beyond the implementation, and could be due to either a lack of sampling or systematic shortcomings in the force field, as was discussed in Huang et al.^30^ For detailed insights into the structural origins of these p*K*_a_ shifts, we refer the reader to the paper of Swails and Roitberg.^19^

The p*K*_a_ values estimated from the Martini 2.0 force field simulations of these proteins do not agree as well with the experiment as those derived from the all-atom simulations (Pearson correlation coefficient (*r*) 0.8 and 0.49; RMSE 0.8 and 2.0 for cardiotoxin V and HEWL, respectively). We speculate that the larger deviation of the p*K*_a_’s in the coarse-grained constant pH simulations could be due to the lower accuracy of the electrostatic interactions. Although we still consider the results obtained with the Martini simulations reasonable, in particular for the peptides, the discrepancies for the titratable residues in proteins suggest that additional parametrization efforts may be required to systematically improve the force field for constant pH MD simulations based on λ-dynamics. Such improvements would be particularly worthwhile considering coarse grained simulations pave the way to perform MD simulations of complete organelles,^75^ in which many processes have a strong pH dependence.

### Efficiency of the Implementation

To demonstrate that linear interpolation of charges is computationally more efficient than the linear interpolation of the potential energy functions for systems with many titratable sites, we investigated how the computational cost of a simulation scales with the number of titratable sites in the system for both approaches. Because we have implemented the interpolation of the charges rather than potential energy functions into the fork of GROMACS 2021 release, whereas the potential energy function interpolation was implemented in a fork of GROMACS 3.3 release, we compare the relative performances of both codes for an increasing number of titratable sites in the system. We define the relative performance as the ratio between the average number of integration steps per time unit for a simulation with constant pH on the one hand and the average number of integration steps per time unit for a normal simulation without constant pH on the other hand.

**Figure 6:**
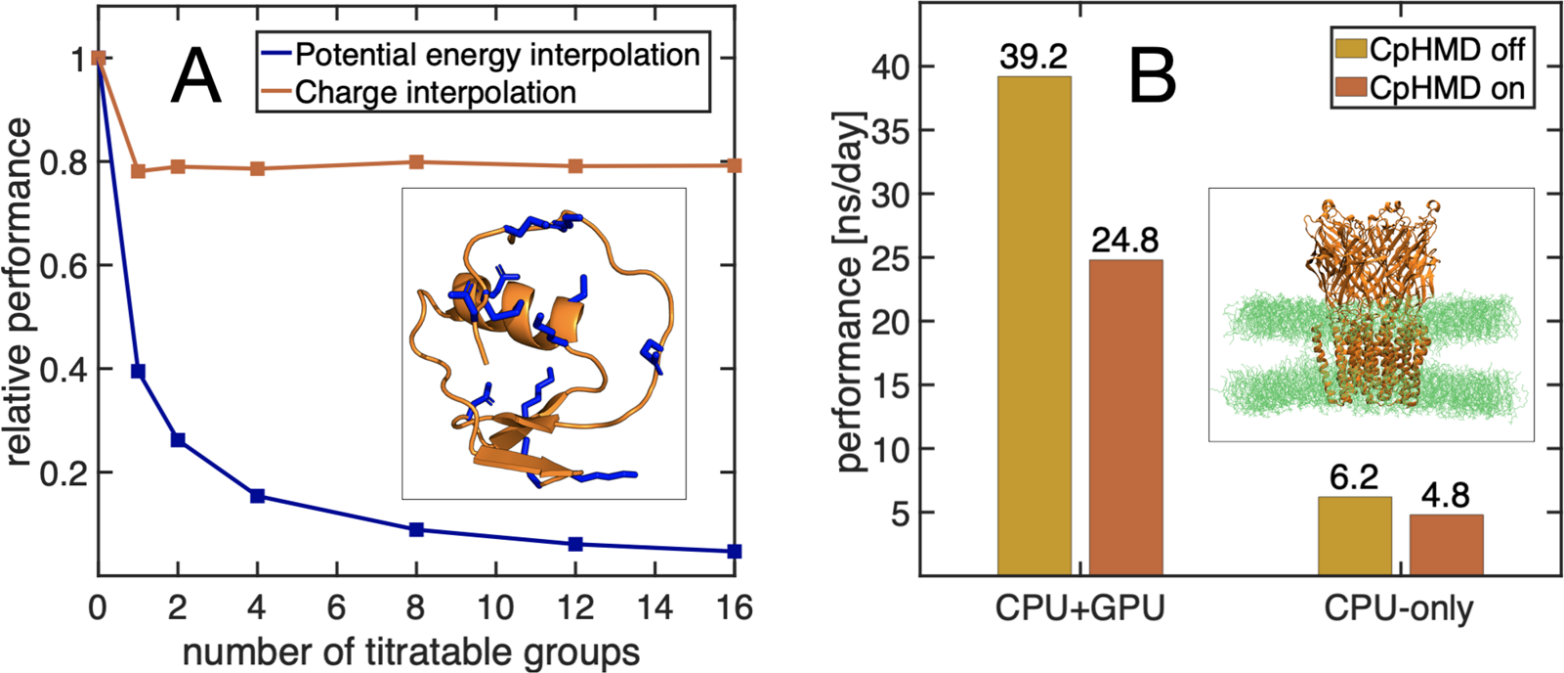
(A) Relative performance of interpolating potentials in a previous implementation of CpHMD into a fork of GROMACS 3.3 release (blue) and of charge interpolation in our new implementation (red) as a function of the number of titratable sites. The simulations were performed for the turkey ovomucoid inhibitor protein (PDB ID: 2GKR(53)), shown in the inset, where the titratable sites are highlighted in stick representation. (B) Comparison of the performance between CPU-only and CPU+GPU implementations for the ligand-gated ion channel GLIC (PDB ID: 4HFI(52)) with 185 titratable sites. In total, the GLIC system contained 292135 atoms.

Figure 6A shows that the relative performance of constant pH simulations with charge interpolation does not decrease when the number of titratable sites included in the simulation increases. Most of the 30–40% drop in performance compared to a normal MD simulation with the same version of GROMACS is caused by the additional calculations and reductions in the nonbonded pair-interaction kernels that are required to obtain the real-space part of the electrostatic potential (Φ(**R**_*i*_, **λ**) in eq 17).

In contrast, the relative performance of constant pH simulations based on the linear interpolation of potential energy functions decreases with the number of titratable sites in the system. This comparison thus demonstrates that by replacing linear interpolation of potentials with linear interpolation of partial charges, we have overcome the major bottleneck in the earlier constant pH implementation in the fork of GROMACS 3.3 release and paved the way toward simulations of large biomolecular systems at constant pH.

An example of such a large system is the proton-gated ion channel GLIC, a membrane protein with 185 titratable residues. Figure 6B shows the performance of the new implementation for this large system when running the simulation on CPU and on a combination of CPU and GPU. While the computational overhead is somewhat larger when using a GPU in addition to a CPU, the overall performance still improves significantly when adding a GPU. We have also implemented a parallel version using MPI. For the GLIC system, the code scales up to 256 cores, on the Mahti supercomputer at CSC, with a performance of 42 ns/day, compared with 61 ns/day without constant pH.

## Conclusions

We have presented and validated a new implementation of λ-dynamics based constant pH molecular dynamics in the GROMACS software. Our implementation combines several developments in this field into a single MD program, including the multisite representation of titratable groups,^46^ charge interpolation,^21^ Particle Mesh Ewald electrostatics,^30^ and charge constraints.^42^ Test calculations on amino acids and proteins suggest that the new implementation is efficient, accurate, and agnostic to force fields. Combined with user-friendly parametrization protocols, presented in the accompanying paper,^55^ we expect that this implementation will pave the way toward routinely including the effect of pH in biomolecular MD simulations.^76^

## Appendix: Constraint Algorithm

We use constraints to restrict sampling to the correct protonation states in the multisite representation as well as to maintain a neutral charge of the simulation box. Both multisite and charge constraints keep the linear combination of a subset of λ-coordinates constant and are applied simultaneously. Thus, there are *N*_*c*_ constraint equations

22

for *k* ≤ *N*_*c*_. Here, **λ** is the vector of all λ_*i*_-coordinates, and *C*^*k*^ is the value of constraint *k*, which can be zero. If σ^*k*^(**λ**) is a multisite constraint, α_*i*_^*k*^ = 1 for λ_*i*_-coordinates that represent one of the protonation states of a residue, while α_*i*_^*k*^ = 0 for all other λ_*i*_-coordinates. If σ^*k*^(**λ**) is a constraint for keeping the overall charge constant, α_*i*_^*k*^ = ∑_*j*_^*N*_atoms_^*i*^^*q*_*j,i*_^*B*^ – *q*_*j,i*_^*A*^, with *N*_atoms_^*i*^ the number of atoms whose charges change as a function of λ_*i*_.

During a leapfrog integration step, all λ_*i*_-coordinates are first propagated without constraints to their unconstrained new values λ_*i*_^*u*^, which do not fulfill the constraints in eq 22. To obtain the constrained λ_*i*_^*c*^ values, we first connect the constrained and unconstrained λ_*i*_ values using the definition of σ^*k*^(**λ**):

23

Because the unconstrained and constrained λ_*i*_-coordinates are also connected by the constraint forces , we have in addition that

24

where ζ^*k*^ is the Lagrange multiplier for constraint *k*, *m*_*i*_ the fictitious mass of λ_*i*_, and Δ*t* the integration time step. Substituting eq 24 in 23 yields

25

which after rearranging can be expressed as

26

The last expression can be rewritten in matrix form

27

where **ζ** = (ζ^1^,···,ζ^*N*_*c*_^)^*T*^ and

28

Because the α_*i*_^*k*^ coefficients remain the same, matrix **A** is computed once at the start of the simulation. At each step, the elements Δ*σ*^*k*^ are evaluated as the difference between the σ^*k*^(**λ**^*u*^) and σ^*k*^(**λ**^*c*^) = *C*^*k*^:

29

The Lagrange multipliers ζ^*k*^ are then obtained from eq 27 and used to correct the unconstrained λ_*i*_ values (eq 24).

## Supporting Information Available

The input files and parameters used for MD simulations in the presented work (ZIP)
A Mathematica notebook with instructions and routines to fit V^MM^ (ZIP)
Description of λ-potentials, the effect of neglecting the interpolation of Lennard-Jones interactions, titration results for Asp and Glu within the single-site representation, a comparison of p*K*_a_ values for HEWL obtained with various λ-dynamics-based constant pH methods, demonstration that charge interpolation requires a single evaluation of the electrostatic potential for both single- and multisite representations (PDF)

## Notes

The fork of GROMACS 2021 with constant pH implemented as described here, is available for download free of charge from https://gitlab.com/gromacs-constantph/constantph. In addition to the source code, also instructions on how to set up and perform MD simulations are available
