# Supplementary material for: Scalable Constant pH Molecular Dynamics in GROMACS
Source: J Chem Theory Comput. 2022 Sep 21;18(10):6148–60. doi: 10.1021/acs.jctc.2c00516 (PMC9558312; doi:10.1021/acs.jctc.2c00516)
Supplement: Supplementary file 3 — ct2c00516_si_003.pdf [file ct2c00516_si_003.pdf]

Supplementary Information: Scalable Constant pH Molecular Dynamics in GROMACS

Noora Aho,[*,†,§] Pavel Buslaev,[*,†,§] Anton Jansen,[‡] Paul Bauer,[‡] Gerrit Groenhof,[*,†] and Berk Hess[*,¶]

†*Nanoscience Center and Department of Chemistry, University of Jyväskylä, Finland*

‡*Department of Applied Physics, Science for Life Laboratory, KTH Royal Institute of Technology, Stockholm, Sweden*

¶*Department of Applied Physics and Swedish e-Science Research Center, Science for Life Laboratory, KTH Royal Institute of Technology, 100 44, Stockholm, Sweden*

§*These authors contributed equally*

E-mail: noora.s.aho@jyu.fi; pavel.i.buslaev@jyu.fi; gerrit.x.groenhof@jyu.fi; hess@kth.se

Contents

1 λ-dependent potential terms **3**

1.1 Biasing and pH-dependent potentials . . . 3

1.2 Partial charges of atoms in different protonation states . . . 5

1.3 Correction potential V^{MM} . . . 6

1.3.1 Correction potential for single site representation . . . 6

1.3.2 Correction potential for multisite representation . . . 6

1.3.3 Coefficients for Asp, Glu and His . . . 11

1.3.4 Using tripeptides as reference compounds . . . 12

2 Effect of Lennard-Jones potential **13**

3 Titrations using single site representation **15**

4 Comparison of HEWL pK_a values to previous calculations **16**

5 Convergence of computed pK_a values **17**

6 Structural analysis of proteins at different pH **20**

6.1 Cardiotoxin V . . . 20

6.2 HEWL . . . 22

7 Residue hydration as a function of pH **24**

8 Charge interpolation for coupled sites **27**

References **30**

1 λ-dependent potential terms

1.1 Biasing and pH-dependent potentials

To enhance sampling at the physically relevant states in the λ-dynamics simulations, we introduce a biasing potential of the form:

$$V_i^{\text{bias}}(\lambda) = -k\left[\exp\left(-\frac{(\lambda-1-b)^2}{2a^2}\right)+\exp\left(-\frac{(\lambda+b)^2}{2a^2}\right)\right]+d\left[\exp\left(-\frac{(\lambda-0.5)^2}{2s^2}\right)\right]$$
$$+0.5w\Big(\Big(1-\text{erf}[r(\lambda+m)]\Big)+\Big(1+\text{erf}[r(\lambda-1-m)]\Big)\Big) \quad \text{(S1)}$$

After providing a value for the barrier height, the eight parameters k, a, b, d, s, w, r and m, are determined in an iterative fashion, as in Donnini *et. al.*[1] Parameters for the default barrier of 7.5 kJ/mol and a barrier of 5.0 kJ/mol are listed in Table S1. The blue curves in Figure S1 illustrate the potential with a barrier of 7.5 kJ/mol.

We also emphasize here, that the iterative procedure to obtain parameters k, a, b, d, s, w, r and m reported in the paper by Donnini *et. al*,[1] differed from the one actually used in both the previous and current implementations. Here, were describe the difference. Parameters d, s, w, r, and m were calculated in the exact same manner, as described in the original paper,[1] however, the parameters k, a, and b were computed differently. Initially, k was set to half of the desired barrier height, a to 0.05, and b to -0.1. Next, parameters k, a, and b were iteratively modified until the terminate conditionals, introduced further, were not satisfied. The iteration loop was organised as follows:

1. The local minimum depth k is updated, so that the total barrier height corresponds to the desired value:

$$k \mathrel{+}= h/2 + \text{Min}_\lambda(V^{\text{bias}}(\lambda)), \quad \text{(S2)}$$

   where h is the desired barrier height

2. The local minimum position b is adjusted:

$$b \mathrel{+}= 0.01 x_0, \tag{S3}$$

where x_0 is the average position of λ:

$$x_0 = \frac{\sum\limits_{\substack{\lambda<0.5 \\ V^{\text{bias}}<0}} \lambda \exp(-V^{\text{bias}}(\lambda))}{\sum\limits_{\substack{\lambda<0.5 \\ V^{\text{bias}}<0}} \exp(-V^{\text{bias}}(\lambda))} \tag{S4}$$

3. The local minimum width a is adjusted:

$$a \;/\!= 1 + 0.01 \frac{\sigma - \sigma_0}{\sigma_0}, \tag{S5}$$

where σ is the dispersion of λ:

$$\sigma = \sqrt{\frac{\sum\limits_{\substack{\lambda<0.5 \\ V^{\text{bias}}<0}} (\lambda - x_0)^2 \exp(-V^{\text{bias}}(\lambda))}{\sum\limits_{\substack{\lambda<0.5 \\ V^{\text{bias}}<0}} \exp(-V^{\text{bias}}(\lambda))}} \tag{S6}$$

and σ_0 is the desired dispersion of λ, which is set to 0.02.

The iterations are repeated until both $\text{Abs}(x_0) < \epsilon$ and $\text{Abs}\left(\frac{\sigma-\sigma_0}{\sigma_0}\right) < \epsilon$ are not satisfied.

The difference with the original routine, described by Donnini *et. al*,[1] is in the requirement for $V^{\text{bias}} < 0$ in equations for x_0 and σ. This requirement is essential for the biasing potential to converge to the desired shape. Additionally, if the desired barrier is smaller than 0.45 kJ/mol, it is always forced to zero.

The pH-dependent potential used in this work is

$$V^{\mathrm{pH}}(\lambda_i) = RT\ln(10)\,[\mathrm{p}K_{\mathrm{a},i} - \mathrm{pH}]\,\frac{1}{1+\exp(-2k_1(\lambda_i - 1 + x_0 s))},\ \text{if pH } > \mathrm{p}K_\mathrm{a}$$
$$V^{\mathrm{pH}}(\lambda_i) = RT\ln(10)\,[\mathrm{p}K_{\mathrm{a},i} - \mathrm{pH}]\,\frac{1}{1+\exp(-2k_1(\lambda_i - x_0))},\ \text{if pH } \le \mathrm{p}K_\mathrm{a}, \quad \text{(S7)}$$

where k_1 and x_0 depend on the parameters r and a of biasing potential (Equation S1). Here, we use $k_1 = 2.5r$ and $x_0 = 2a$. This pH potential, as well as the combination with the biasing potential is plotted in Figure S1 for two pH values

Table S1: Parameters for the double well biasing potential for barrier heights 5.0 kJ/mol and 7.5 kJ/mol.

	k	a	b	d	s	w	r	m
barrier 5.0 kJ/mol	3.1889	0.0363	0.0044	2.50	0.30	1000.0	13.5	0.2019
barrier 7.5 kJ/mol	4.7431	0.0435	0.0027	3.75	0.30	1000.0	13.5	0.2019

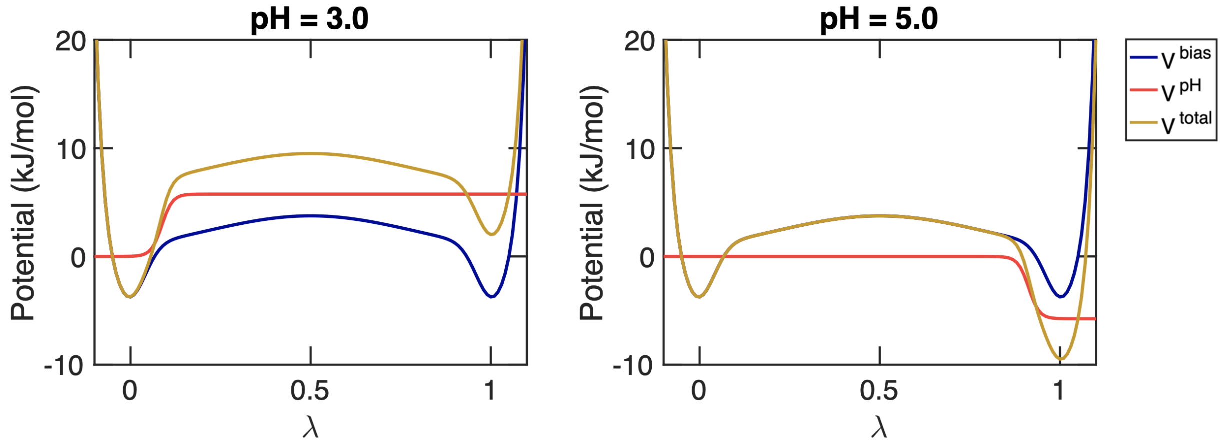

Figure S1: Combination of biasing and pH potentials at pH=3.0 (left) and pH=5.0 (right), when pK_a=4.0. The blue profile shows the biasing potential V^{bias} (Equation S1) with a barrier height of 7.5 kJ/mol. The red profile shows the pH-dependent potential V^{pH} (Equation S7). The yellow profile shows the sum of the two potentials $V^{\mathrm{total}} = V^{\mathrm{bias}} + V^{\mathrm{pH}}$

1.2 Partial charges of atoms in different protonation states

Default partial charges for the atoms in the side chains of Asp, Glu, and His were used to model the electrostatic interactions of these residues in their different protonation states

for constant pH simulations with both the CHARMM36 and Martini 2.0 force fields. These charges are provided via a separate `coefficients.dat` file that contains for each amino acid a list of atoms with their charges in the various protonation states. This file is included in the supplementary archive `SI_constant_ph_gromacs.zip`.

1.3 Correction potential V^{MM}

The quantum mechanical contributions to proton binding that are missing from classical molecular mechanics force fields are compensated for by a correction potential V^{MM}. To propagate λ-coordinates we only need the gradients of this potential with respect to the λ-coordinates. Analytical expressions for these gradients are obtained as polynomial fits to the derivatives of the reference free energy along the charge interpolation path. Below we provide the details for the fitting of $\partial V^{\mathrm{MM}}/\partial\lambda$ for both the single-site and multisite representations of a titratable residue.

1.3.1 Correction potential for single site representation

If the single site representation is used, V^{MM} is obtained as follows: First, the trajectory averages of $\langle \frac{\partial V}{\partial \lambda} \rangle_\lambda$ are computed at various values of λ between -0.1 and 1.1. In our implementation, we can accumulate $\langle \frac{\partial V}{\partial \lambda} \rangle_\lambda$ at fixed values of λ by setting the fictitious mass of the λ-particle to zero. Subsequently, $\partial V^{\mathrm{MM}}/\partial\lambda$ is obtained as a polynomial fit to the $\langle \frac{\partial V}{\partial \lambda} \rangle_\lambda$ values.

1.3.2 Correction potential for multisite representation

The correction potential for the multisite representation with n protonation states is obtained by performing a multi-dimensional fit to the trajectory averages of $\langle \frac{\partial V}{\partial \lambda_k} \rangle_{(\lambda_1^{(\alpha)}, \lambda_2^{(\alpha)}, \ldots, \lambda_n^{(\alpha)})}$. The N^{grid} grid points $(\lambda_1^{(\alpha)}, \lambda_2^{(\alpha)}, \ldots, \lambda_n^{(\alpha)})$ for $1 \le \alpha \le N^{\mathrm{grid}}$, are selected under the constraint

$$\sum_{j=1}^{n} \lambda_j^{(\alpha)} = 1, \tag{S8}$$

We represent the correction potential by a $(k+1)^{\text{th}}$ order polynomial

$$V^{\text{MM}}(\lambda_1, \lambda_2, ..., \lambda_n) = \sum_{t=0}^{k+1} \left[\sum_{\substack{\sum_{i=1}^{n} p_i = t \\ p_i \geq 0}} a_{p_1,p_2,...,p_n} \prod_{i=1}^{n} (\lambda_i)^{p_i} \right] \tag{S9}$$

where p_i indicates the power of λ_i. The derivative of this polynomial with respect to λ_j is of order k:

$$\frac{\partial V^{\text{MM}}}{\partial \lambda_j}(\lambda_1, \lambda_2, ..., \lambda_n) = \sum_{t=1}^{k+1} \left[\sum_{\substack{\sum_{i=1}^{n} p_i = t \\ p_{i,i\neq j} \geq 0 \\ p_j \geq 1}} p_j a_{p_1,p_2,...,p_n} (\lambda_j)^{p_j - 1} \prod_{\substack{i=1 \\ i \neq j}}^{n} (\lambda_i)^{p_i} \right] \tag{S10}$$

Because of the constraint (Equation S8), we can substitute $\lambda_n = 1 - \sum_1^{n-1} \lambda_i$ to get

$$\frac{\partial V^{\text{MM}}}{\partial \lambda_j}(\lambda_1, \lambda_2, ..., \lambda_n) =$$

$$\begin{cases} \sum_{t=1}^{k+1} \left[\sum_{\substack{\sum_{i=1}^{n} p_i = t \\ p_{i,i\neq j} \geq 0 \\ p_j \geq 1}} \left\{ p_j a_{p_1,p_2,...,p_n} (\lambda_j)^{p_j-1} \left(\prod_{\substack{i=1 \\ i\neq j}}^{n-1} (\lambda_i)^{p_i} \right) \left(1 - \sum_{r=1}^{n-1} \lambda_r \right)^{p_n} \right\} \right], & \text{if } k \neq n \\ \sum_{t=1}^{k+1} \left[\sum_{\substack{\sum_{i=1}^{n} p_i = t \\ p_{i,i<n} \geq 0 \\ p_n \geq 1}} \left\{ p_n a_{p_1,p_2,...,p_n} \left(1 - \sum_{r=1}^{n-1} \lambda_r \right)^{p_n - 1} \left(\prod_{i=1}^{n-1} (\lambda_i)^{p_i} \right) \right\} \right], & \text{if } k = n \end{cases}$$

$$= \sum_{t=0}^{k} \left[\sum_{\substack{\sum_{i=1}^{n-1} q_i = t \\ q_i \geq 0}} b^{t,j}_{q_1,q_2,...,q_{n-1}} \prod_{i=1}^{n-1} (\lambda_i)^{q_i} \right] \tag{S11}$$

where we have introduced the coefficients $b^{t,j}_{q_1,q_2,...,q_{n-1}}$ as short-hand notations for the linear combinations of $a_{p_1,p_2,...,p_n}$:

$$b^{t,j}_{q_1,q_2,...,q_{n-1}} = \sum_{r=0}^{r=k+1}\left[\sum_{\sum_{i=1}^{n} p_i = r} m^{t,j,q_1,...,q_{n-1}}_{p_1,p_2,...,p_n} a_{p_1,p_2,...,p_n}\right] \tag{S12}$$

where $m^{t,j,q_1,...,q_{n-1}}_{p_1,p_2,...,p_n}$ are the expansion coefficients obtained by contracting the terms between brackets in Equation S11. To simplify notation, we write expression S12 in matrix form:

$$\mathbf{b} = \mathbf{M}\mathbf{a}, \tag{S13}$$

where

$$\mathbf{M} = \begin{pmatrix} m^{k,1,k,0,...,0}_{k+1,0,...,0} & m^{k,1,k,0,...,0}_{k,1,...,0} & \cdots & m^{k,1,k,0,...,0}_{k,0,...,0} & \cdots & m^{k,1,k,0,...,0}_{0,0,...,0} \\ m^{k,1,k-1,1,...,0}_{k+1,0,...,0} & m^{k,1,k-1,1,...,0}_{k,1,...,0} & \cdots & m^{k,1,k-1,1,...,0}_{k,0,...,0} & \cdots & m^{k,1,k-1,1,...,0}_{0,0,...,0} \\ \vdots & \vdots & & \ddots & & \vdots \\ m^{k-1,1,k-1,0,...,0}_{k+1,0,...,0} & m^{k-1,1,k-1,0,...,0}_{k,1,...,0} & \cdots & m^{k-1,1,k-1,0,...,0}_{k,0,...,0} & \cdots & m^{k-1,1,k-1,0,...,0}_{0,0,...,0} \\ \vdots & \vdots & & \ddots & & \vdots \\ m^{0,n,0,0,...,0}_{k+1,0,...,0} & m^{0,n,0,0,...,0}_{k,1,...,0} & \cdots & m^{0,n,0,0,..,0}_{k,0,...,0} & \cdots & m^{0,n,0,0,...,0}_{0,0,...,0} \end{pmatrix} \tag{S14}$$

and $\mathbf{a} = (a_{k+1,0,...,0}, a_{k,1,...,0}, ..., a_{0,0,...,0})^T$.

The best multi-dimensional fit for $\partial V^{\mathrm{MM}}/\partial\lambda_k$ is obtained by finding the coefficients $b^{t,j}_{q_1,q_2,...,q_{n-1}}$ that minimize the squared deviation between the $\partial V^{\mathrm{MM}}(\lambda_1, ..., \lambda_n)/\partial\lambda_k$ and the average value of $\langle\partial V/\partial\lambda_k\rangle_{(\lambda_1^{(\alpha)},\lambda_2^{(\alpha)},...,\lambda_n^{(\alpha)})}$ over all grid points α (*i. e.*, $(\lambda_1^{(\alpha)}, \lambda_2^{(\alpha)}, ..., \lambda_n^{(\alpha)})$):

$$\hat{\mathbf{b}} = \underset{\mathbf{b}}{\operatorname{argmin}}\, S(\mathbf{b}) \tag{S15}$$

where $\mathbf{b} = \left(b^{k,1}_{k,0,...,0}, b^{k,1}_{k-1,1,...,0}, ..., b^{k,1}_{0,0,...,k}, b^{k-1,1}_{k-1,0,...,0}, b^{k-1,1}_{k-2,1,...,0}, ..., b^{k-1,1}_{0,0,...,k-1}, ..., b^{0,n}_{0,0,...,0}\right)^T$, $\hat{\mathbf{b}}$ is the optimal solution, and $S(\mathbf{b})$ is the sum of squared differences between $\partial V^{\mathrm{MM}}/\partial\lambda_j$ and the

value of $\langle \partial V/\partial \lambda_j \rangle_{(\lambda_1^{(\alpha)},\lambda_2^{(\alpha)},\ldots,\lambda_n^{(\alpha)})}$ at the grid points:

$$S(\mathbf{b}) = \sum_{j=1}^{n} \sum_{i} \left(\left\langle \frac{\partial V}{\partial \lambda_j} \right\rangle_{(\lambda_1^{(\alpha)},\lambda_2^{(\alpha)},\ldots,\lambda_n^{(\alpha)})} - \sum_{\sum_{l=1}^{n-1} q_l <= k} b_{q_1,q_2,\ldots,q_{n-1}}^{k,j} \prod_{l=1}^{n-1} (\lambda_l^{(\alpha)})^{q_l} \right)^2 \tag{S16}$$

With the average values of $\langle \frac{\partial V}{\partial \lambda_j} \rangle_{(\lambda_1^{(\alpha)},\lambda_2^{(\alpha)},\ldots,\lambda_n^{(\alpha)})}$ at the grid points obtained from the trajectories, the residual sum of squares $S(\mathbf{b})$ can be cast in matrix notation:

$$S(\mathbf{b}) = \|\mathbf{f} - \mathbf{X}\mathbf{b}\|^2 , \tag{S17}$$

where $\|...\|$ denotes the Euclidean norm, and $\mathbf{f}$ and $\mathbf{X}$ are:

$$\mathbf{f} = \begin{pmatrix} \langle \frac{\partial V}{\partial \lambda_1} \rangle_{\left(\lambda_1^{(1)},\lambda_2^{(1)},\ldots,\lambda_n^{(1)}\right)} \\ \langle \frac{\partial V}{\partial \lambda_1} \rangle_{\left(\lambda_1^{(2)},\lambda_2^{(2)},\ldots,\lambda_n^{(2)}\right)} \\ \vdots \\ \langle \frac{\partial V}{\partial \lambda_1} \rangle_{\left(\lambda_1^{\left(N^{\text{grid}}\right)},\lambda_2^{\left(N^{\text{grid}}\right)},\ldots,\lambda_n^{\left(N^{\text{grid}}\right)}\right)} \\ \langle \frac{\partial V}{\partial \lambda_2} \rangle_{\left(\lambda_1^{(1)},\lambda_2^{(1)},\ldots,\lambda_n^{(1)}\right)} \\ \langle \frac{\partial V}{\partial \lambda_2} \rangle_{\left(\lambda_1^{(2)},\lambda_2^{(2)},\ldots,\lambda_n^{(2)}\right)} \\ \vdots \\ \langle \frac{\partial V}{\partial \lambda_2} \rangle_{\left(\lambda_1^{\left(N^{\text{grid}}\right)},\lambda_2^{\left(N^{\text{grid}}\right)},\ldots,\lambda_n^{\left(N^{\text{grid}}\right)}\right)} \\ \vdots \\ \langle \frac{\partial V}{\partial \lambda_n} \rangle_{\left(\lambda_1^{(1)},\lambda_2^{(1)},\ldots,\lambda_n^{(1)}\right)} \\ \langle \frac{\partial V}{\partial \lambda_n} \rangle_{\left(\lambda_1^{(2)},\lambda_2^{(2)},\ldots,\lambda_n^{(2)}\right)} \\ \vdots \\ \langle \frac{\partial V}{\partial \lambda_n} \rangle_{\left(\lambda_1^{\left(N^{\text{grid}}\right)},\lambda_2^{\left(N^{\text{grid}}\right)},\ldots,\lambda_n^{\left(N^{\text{grid}}\right)}\right)} \end{pmatrix} \qquad \mathbf{X} = \begin{pmatrix} \mathbf{X}^{\text{single}} & \mathbf{0} & \mathbf{0} & \cdots & \mathbf{0} \\ \mathbf{0} & \mathbf{X}^{\text{single}} & \mathbf{0} & \cdots & \mathbf{0} \\ \vdots & \vdots & \vdots & \ddots & \vdots \\ \mathbf{0} & \mathbf{0} & \mathbf{0} & \cdots & \mathbf{X}^{\text{single}} \end{pmatrix} \tag{S18}$$

Here, $\mathbf{0}$ is a 0-filled matrix with the same dimensions as $\mathbf{X}^{\text{single}}$, which is defined by:

$$\mathbf{X}^{\text{single}} = \begin{pmatrix} \Lambda_1^1 & \Lambda_1^2 & \cdots & \Lambda_1^{n_c} \\ \Lambda_2^1 & \Lambda_2^2 & \cdots & \Lambda_2^{n_c} \\ \vdots & \vdots & \ddots & \vdots \\ \Lambda_{N^{\text{grid}}}^1 & \Lambda_{N^{\text{grid}}}^2 & \cdots & \Lambda_{N^{\text{grid}}}^{n_c} \end{pmatrix}, \tag{S19}$$

where Λ_α^j is the value of $\prod_{l=1}^{n-1}(\lambda_l^{(\alpha)})^{q_l}$ at α-th grid point for j-th element of the set of powers q_l. The polynomial representing the $\partial V^{\text{MM}}/\partial\lambda_k$ in Equation S11 is a linear combination of n_c $\prod_{l=1}^{n-1}(\lambda_l^{(\alpha)})^{q_l}$ terms. Each of these terms is defined by a combination of powers q_l and each set of powers fulfills the inequality:

$$\sum_{l=1}^{n-1} q_l \leq k, \quad q_l \geq 0, \ l \in \{1, 2, \ldots, n-1\}$$

However, there can be combinations of k and n for which the coefficients $b^{t,j}_{q_1,q_2,\ldots,q_{n-1}}$ are linearly dependent and the rank of $\mathbf{M}$ is smaller than the number of rows in $\mathbf{M}$ (Equation S13). In such situations, the total number of linearly independent combinations of the coefficients $b^{t,j}_{q_1,q_2,\ldots,q_{n-1}}$ is rank($\mathbf{M}$). For the $n_c * n - \text{rank}(\mathbf{M})$ linear combinations, we have

$$\sum_i \epsilon_i^l M_{ij} = (0, 0, ..., 0), \tag{S20}$$

where l is an index for each of the $n_c * n - \text{rank}(\mathbf{M})$ linearly dependent combinations with $1 \leq l \leq n_c * n - \text{rank}(\mathbf{M})$; and ϵ_i^l are the expansion coefficients for these combinations. In matrix notation

$$\mathbf{Q}^T\mathbf{M} = \mathbf{0}, \tag{S21}$$

with $\mathbf{Q}$ the full-rank matrix of the coefficients ϵ_i^l. If rank($\mathbf{Q}$) $= 0$, there are no linear dependencies between the coefficients $b^{t,j}_{q_1,q_2,\ldots,q_{n-1}}$ and the solution for the optimization problem

S15 is obtained as

$$\hat{\mathbf{b}} = \left(\mathbf{X}^T\mathbf{X}\right)^{-1}\mathbf{X}^T\mathbf{f}, \tag{S22}$$

where $\mathbf{X}^T\mathbf{X}$ is considered invertible,[2] which will always be the case if the number of grid points (N^{grid}) is sufficiently large for a given maximum order of the polynomial. If $\text{rank}(\mathbf{Q}) > 0$, the optimization in S15 transforms into a constrained estimation. From equations S13 and S21 the constraints on the estimator $\mathbf{b}$ can be written as

$$\mathbf{Q}^T\mathbf{b} = \begin{pmatrix} 0 \\ 0 \\ \vdots \\ 0 \end{pmatrix} \tag{S23}$$

and the solution for optimal estimator is given by[2]

$$\hat{\mathbf{b}} = \mathbf{R}\left(\mathbf{R}^T\mathbf{X}^T\mathbf{X}\mathbf{R}\right)^{-1}\mathbf{R}^T\mathbf{X}^{-1}\mathbf{f}, \tag{S24}$$

where $\mathbf{R}$ is a matrix such that $\mathbf{R}^T\mathbf{Q} = \mathbf{0}$ and $\text{rank}\left[(\mathbf{Q}\ \mathbf{R})\right] = n_c * n$. $\mathbf{R}$ is obtained by computing the basis set of complement space of $\mathbf{Q}$.

1.3.3 Coefficients for Asp, Glu and His

The procedure for obtaining the gradients of force field free energy associated with deprotonation of the amino acids in their reference states is explained in the Methods section of the main text. The coefficients obtained by applying the fitting procedures described above to these gradients are provided as input and are listed in `coefficients.dat` file that is included in the supporting archive `SI_constant_ph_gromacs.zip`.

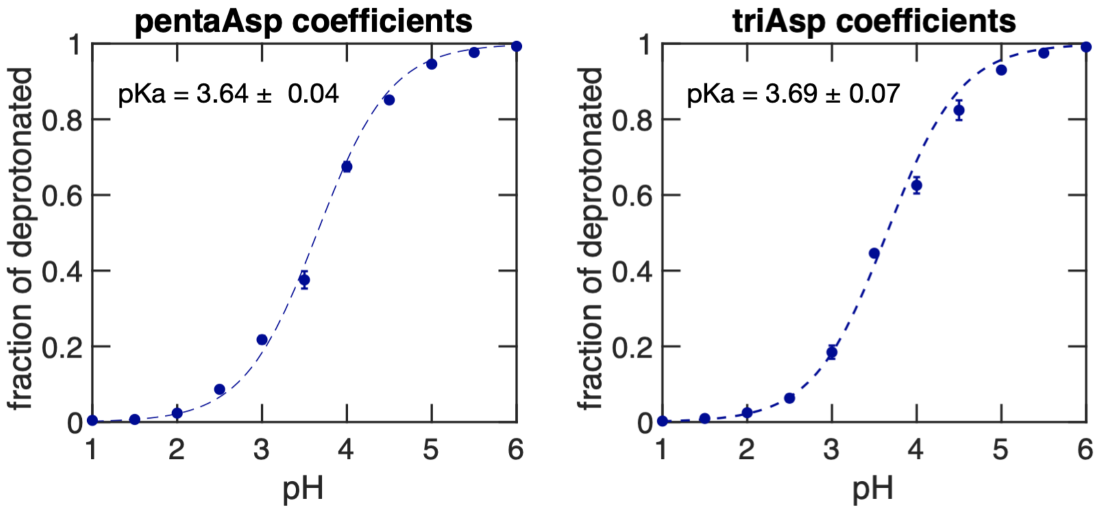

Figure S2: Titration curves for tripeptide Asp using $\partial V^{\mathrm{MM}}/\partial\lambda$ coefficients obtained for pentaAsp (on the left) and triAsp (on the right). Dots show the fraction of deprotonated acid from CpHMD simulations, and dashed lines are the theoretical curve for reference $\mathrm{p}K_{\mathrm{a}} = 3.65$.

1.3.4 Using tripeptides as reference compounds

In this work, we performed both the thermodynamic integration runs for obtaining V^{MM} and the test simulations for tripeptides, whereas the experimental reference $\mathrm{p}K_{\mathrm{a}}$ values for the amino acids were obtained for pentapeptides.[3,4] We validated this choice by computing also the titration curve for the Asp tripeptide using coefficients obtained from thermodynamic integration runs on a pentapeptide system. As shown in Figure S2, the titration curves are virtually identical, suggesting that the correction potentials obtained from the calibration runs on the tripeptides, are transferable.

2 Effect of Lennard-Jones potential

In this work, we interpolated the charges between protonation states, but not the Lennard-Jones parameters. The motivation for this choice is twofold: First, while implementing the interpolations is conceptually straightforward, re-organizing GROMACS to realize such interpolations is very time-consuming. Second, the contribution to the proton affinity due to changes in the Lennard-Jones interactions between the protonation states is much smaller than the contribution from the Coulomb interactions.[5] Therefore, we consider it a reasonable approximation to neglect the effect of the changes in Lennard-Jones parameters.

To verify the validity of this approximation for the two force fields used in this work, we computed the change in free energy associated with the deprotonation of Asp in the tripeptide test system and of Asp-59 in Cardiotoxin V. To test the influence of the various interactions, we performed thermodynamic integration (TI) runs, in which we interpolated:

1. all interactions, including bonded, Coulomb and Lennard-Jones interactions
2. only the electrostatic interactions
3. the electrostatic and Lennard-Jones interactions
4. the electrostatic and bonded interactions

Because in the Martini 2.0 force field, the bonded interactions do not change upon deprotonation, we only compared 2 and 3 for Martini.

We interpolated the interactions in 21 equidistant steps between $\lambda = 0$ and $\lambda = 1$. Each step was simulated for 51 ns, of which the first nanosecond was used for equilibration and hence discarded from the analysis. We used Bennett's acceptance ratio method[6] to extract the free energies. In Figure S3, we plot $\Delta\Delta G$, defined as the difference between the free energy differences (ΔG) associated with deprotonating Asp in the protein and in the peptide, for the four TI runs. While the contribution of interpolating the Lennard-Jones to the $\Delta\Delta G$ values is below 10% for both the CHARMM36 AA and Martini 2.0 CG force field,

the contribution of interpolating also the bonded interactions is of the same order but of opposing sign. We, therefore, conclude that for the force fields used in this work neglecting the interpolation of both bonded and Lennard-Jones interactions and only interpolate the charges, provides a sufficiently accurate description of the effect of the environment on the proton affinities.

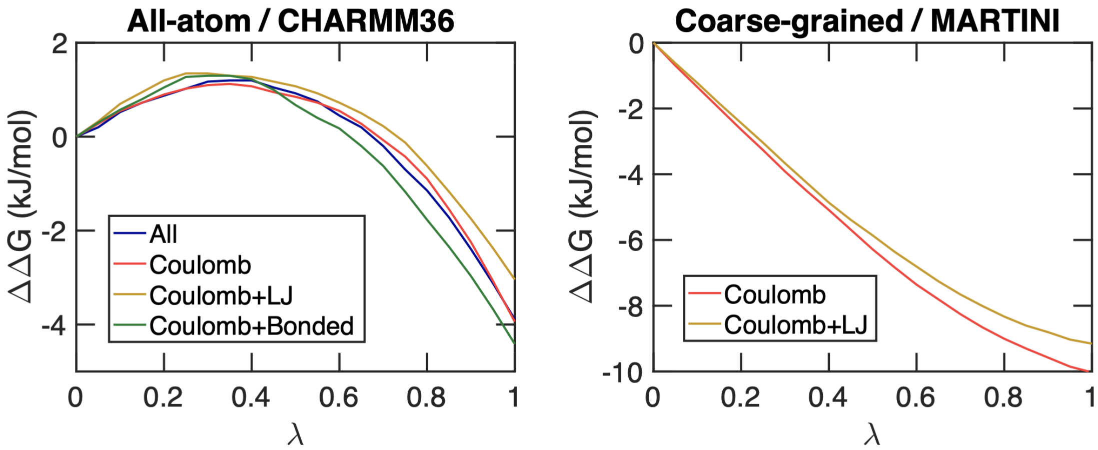

Figure S3: Difference between the free energy difference associated with deprotonation of aspartic acid in cardiotoxin V and in the tripeptide (*i.e.*, $\Delta\Delta G$), for both the AA CHARMM36 and CG Martini force fields, when interpolating all interactions, only Coulomb interactions, Coulomb plus Lennard-Jones interactions, and Coulomb plus bonded interactions.

3 Titrations using single site representation

The all-atom titration simulations presented in the main text were performed with the multisite representation for all amino acids. While for His the multisite representation is necessary, the Asp and Glu side chains could have been modeled with the singe-site representation as well. To demonstrate that this choice is not relevant for the results of the simulations, we repeated the titration simulations with the single-site representation for the tripeptides, keeping all other simulation parameters the same. Comparing the titration curves obtained with the single-site representation in Figure S4 to the curves obtained with the multisite representation in Figure 3 of the main text, we conclude that both representations yield identical results.

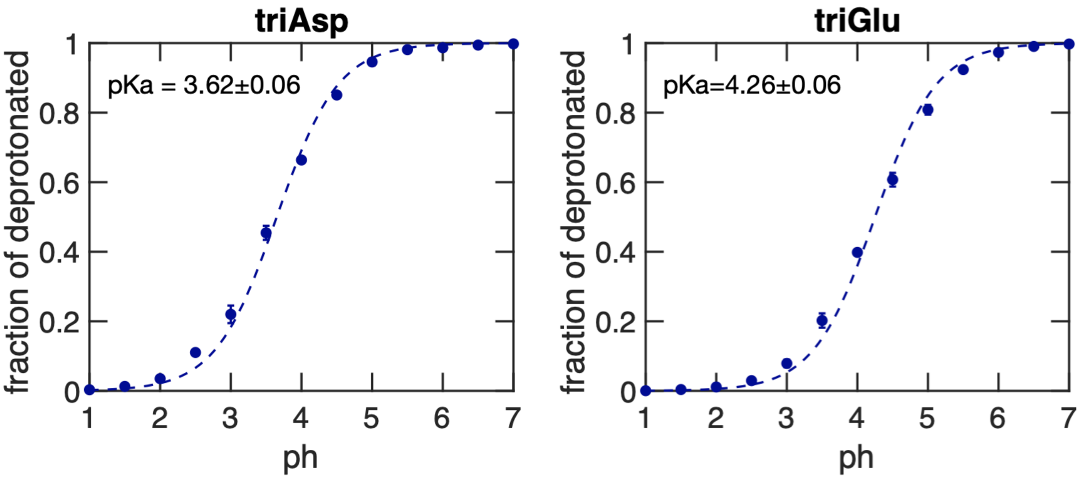

Figure S4: Titration curves of tripeptides Glu and Asp in water, using AA CHARMM36 force field in the single site representation. Charge constraints, in combination with 20 buffer particles were used to keep the box neutral in all simulations. Dots show the fraction of frames in which the residue is deprotonated, and the dashed lines represent the fits to the Henderson-Hasselbalch equation. From these fits the pK_a values were estimated and included inside the graphs.

4 Comparison of HEWL pK_a values to previous calculations

The scatter plot in Figure S5 compares the calculated pK_a values for HEWL obtained in this work to those obtained by others,[7,8] as well as to experiment.[9]

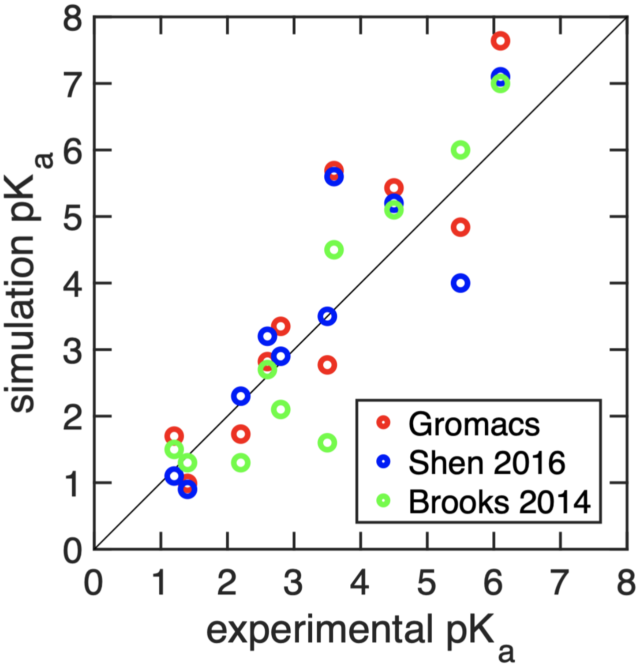

Figure S5: Correlation between the experimental and calculated pK_a for the HEWL protein. Red circles show the results obtained with our implementation. Blue circles are the results published by Shen *et.al.*[7] Green circles are results published by Brooks *et.al.*[8] Experimental pK_a values were taken from Webb *et al.*[9]

5 Convergence of computed pK_a values

To monitor the convergence of the predicted pK_a values in cardiotoxin V and HEWL, we computed the cumulative average of the pK_a values (figure S6 and S8) and S^{deprot} (figure S7 and S9) at different pH values as a function of simulation time window. The time window over which S^{deprot} and pK_a were averaged was increased from 5 to 50 ns in steps of 5 ns. The results, averaged over the replicas, are shown in Figures S6-S7 for Cardiotoxin and S8-S9 for HEWL.

Within 50 ns all pK_a values, as well as S^{deprot} at high, average, and low pH values level off, indicating convergence. Because S^{deprot} converges slower than the pK_a values, convergence of the latter is no proof that the simulations are converged. The slow convergence of protonation states of Asp52-Glu35 pair in HEWL has been observed by others, who also discussed the origin for the slow convergence as well.[7,10]

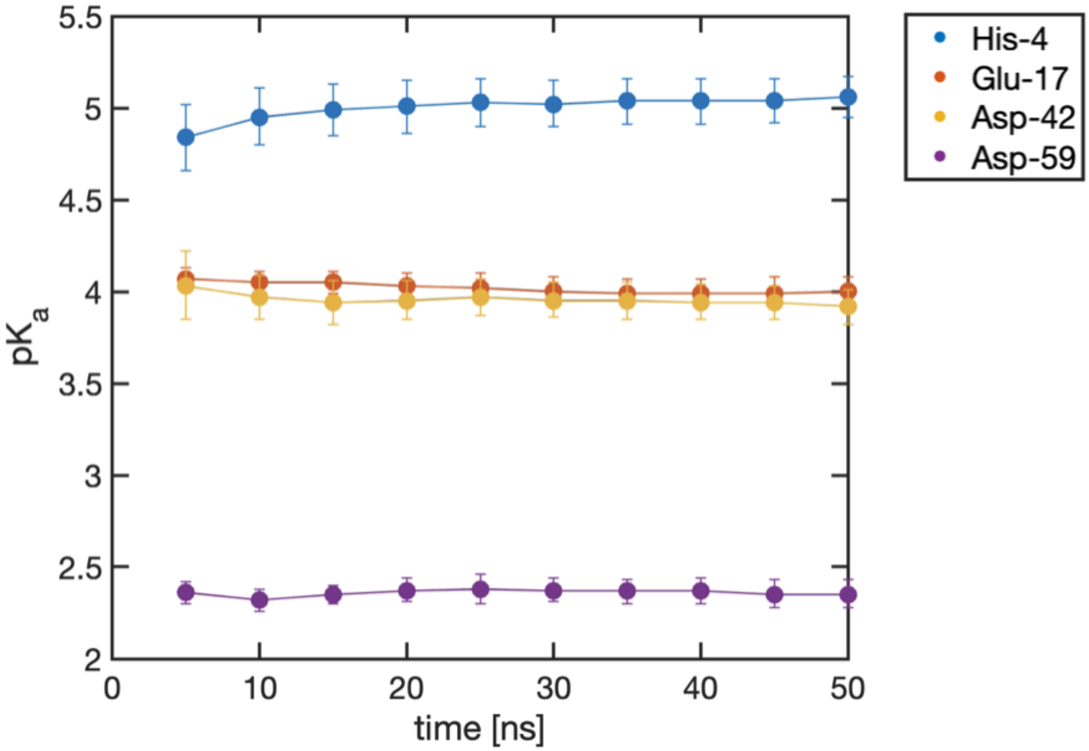

Figure S6: Time evolution of the cumulative pK_a values in cardiotoxin V.

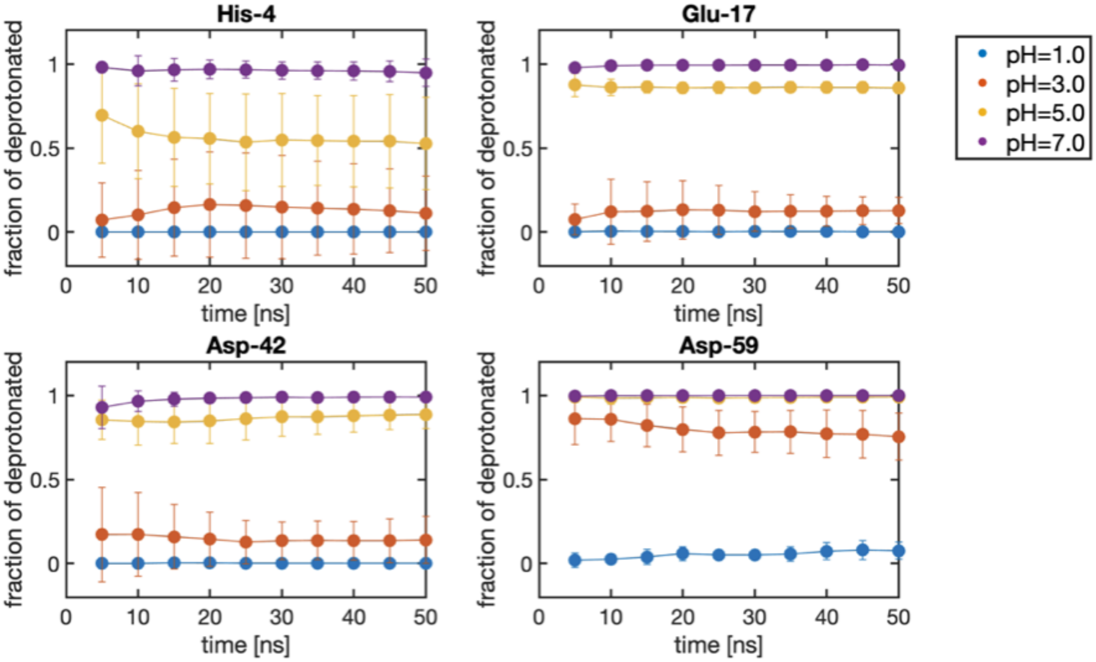

Figure S7: Time evolution of the cumulative average of S^{deprot} in cardiotoxin V at different pH values.

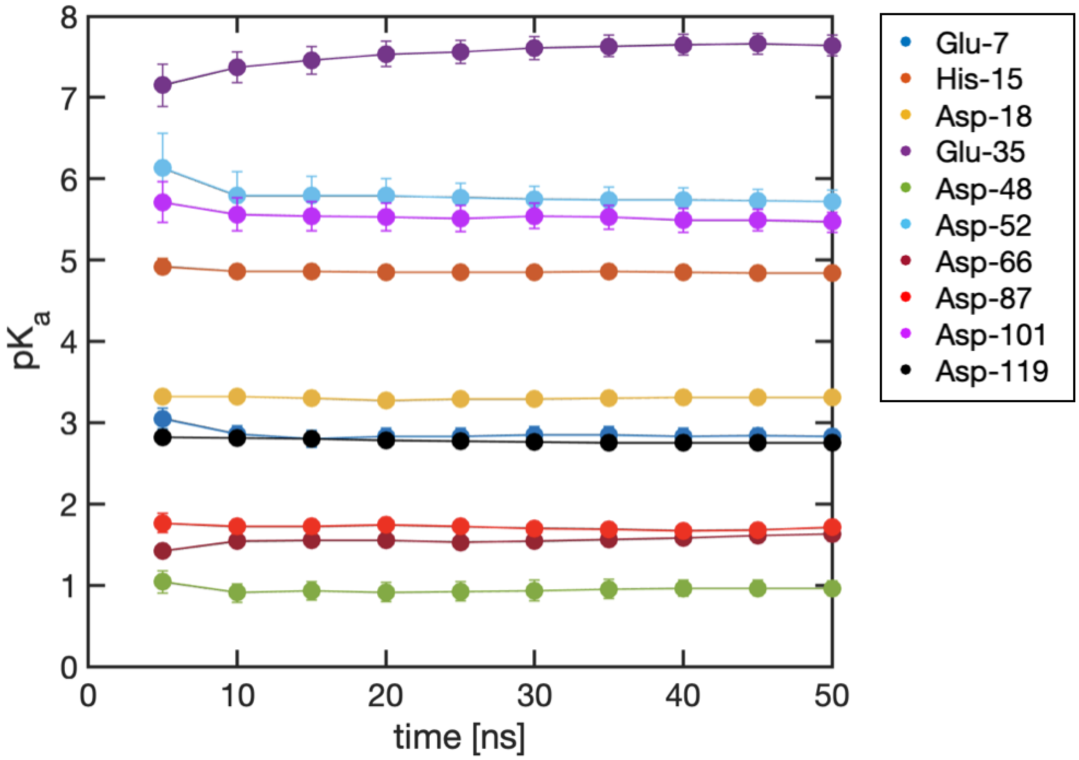

Figure S8: Time evolution of the cumulative pK_{a} values in HEWL.

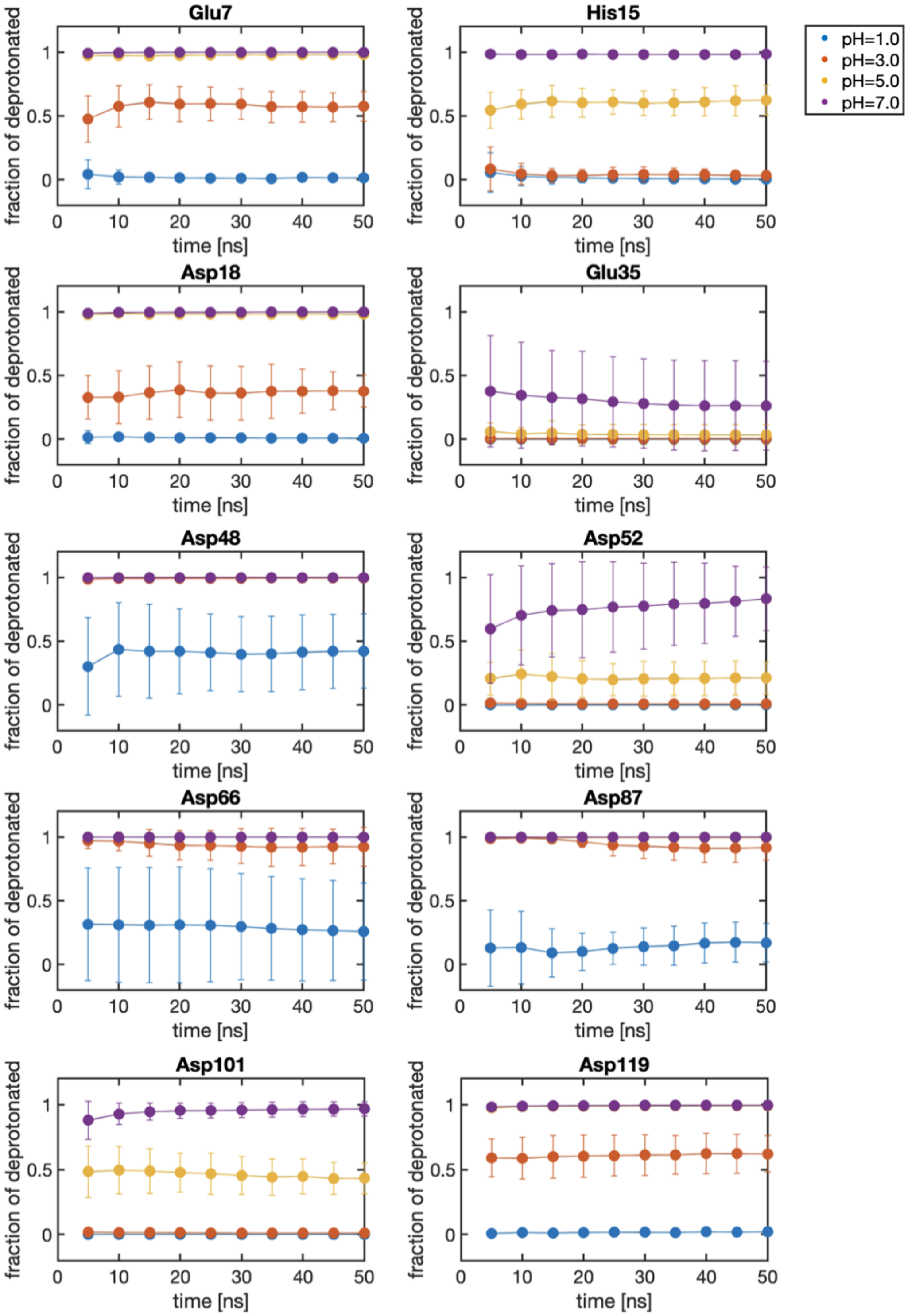

Figure S9: Time evolution of the cumulative average of S^{deprot} in HEWL at different pH values.

6 Structural analysis of proteins at different pH

6.1 Cardiotoxin V

The effect of pH on the structure of cardiotoxin V is moderate. Overall, the protein is stable over the whole pH range, and no major conformational changes are observed (figure S10,S11).

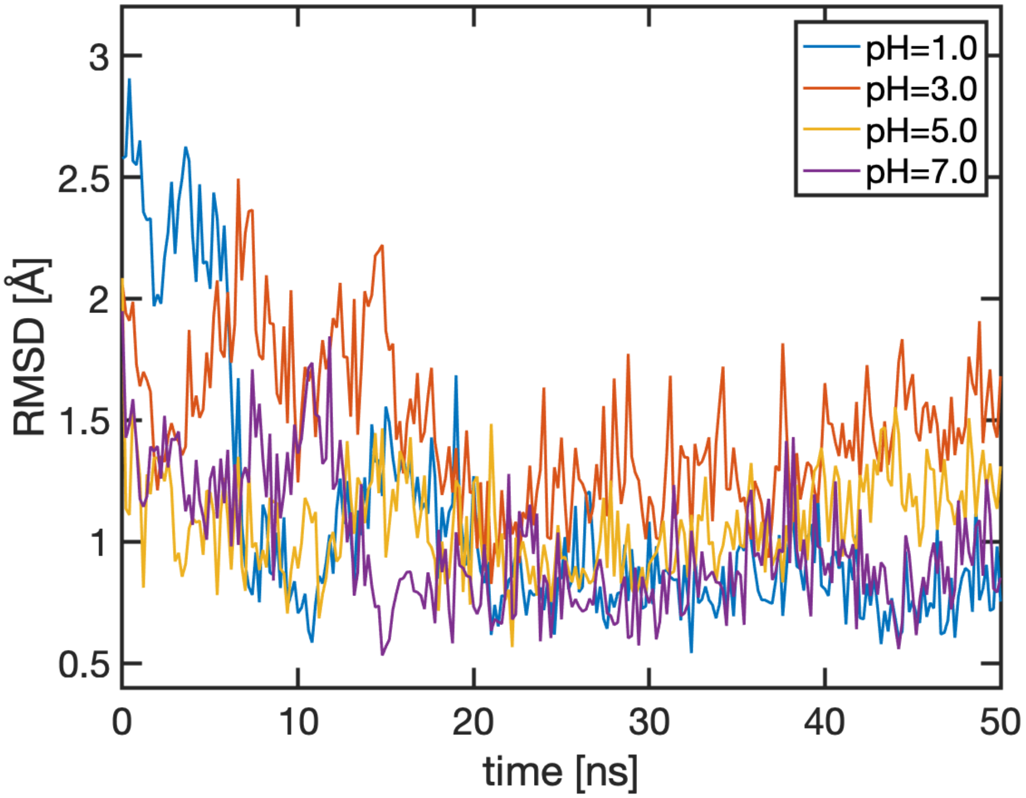

Figure S10: RMSD of cardiotoxin V with respect to average structure at low, average and high pH. RMSD was computed for C_α atoms.

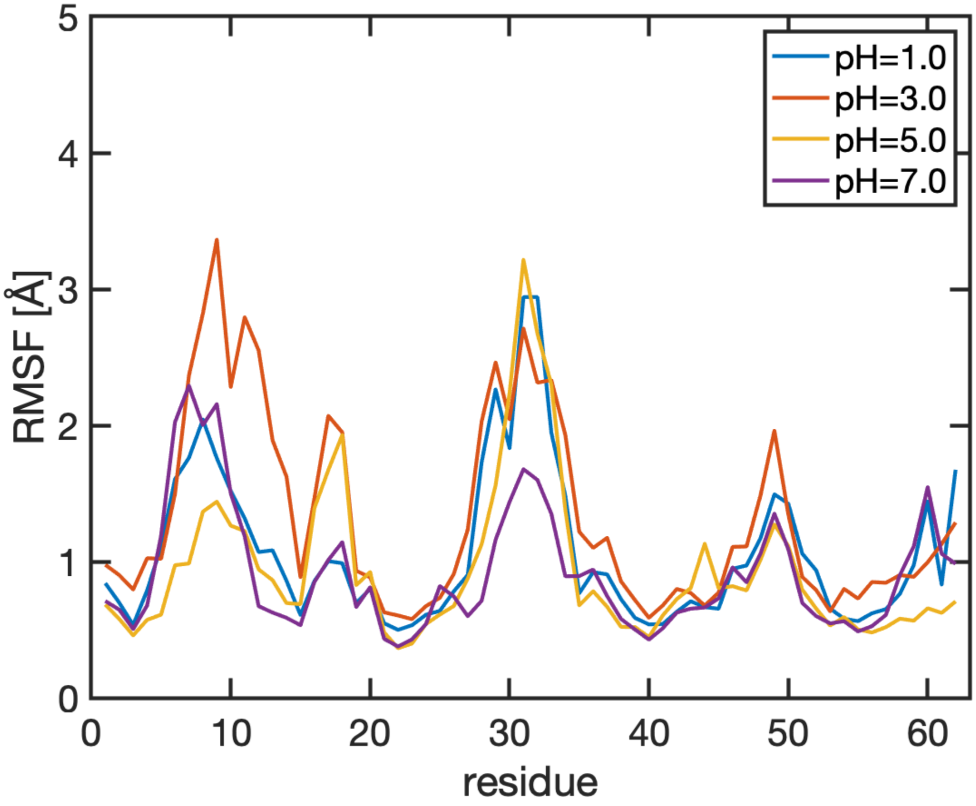

Figure S11: RMSF of cardiotoxin V residues with respect to average structure at low, average and high pH. RMSF was computed for C_α atoms.

6.2 HEWL

The effect of pH on the structure of HEWL is moderate. Overall, the protein is stable over the whole pH range, and no major conformational changes are observed (figure S12,S13). The major effect of pH on the structure is observed in loops formed by residues 40-50 and 65-75. The flexibility of these loops increases with decreasing pH. These trends are in line with previous findings for HEWL and have been discussed in detail elsewhere.[10]

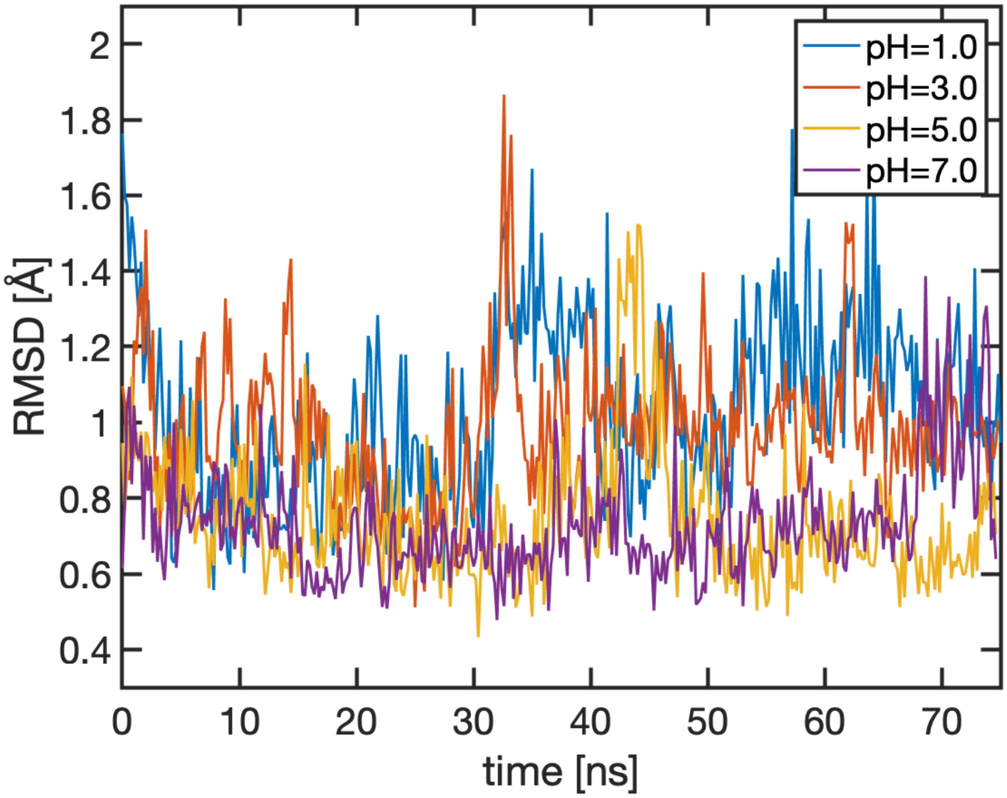

Figure S12: RMSD of HEWL with respect to average structure at low, average and high pH. RMSD was computed for C_α atoms.

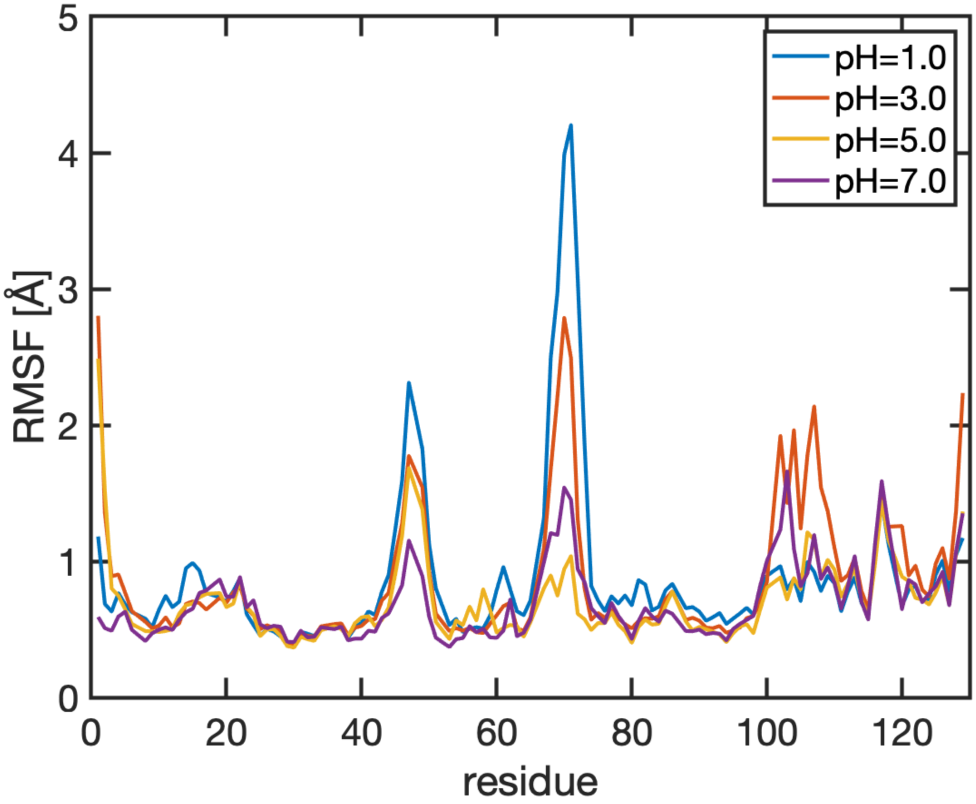

Figure S13: RMSF of HEWL residues with respect to average structure at low, average and high pH. RMSF was computed for C_α atoms.

7 Residue hydration as a function of pH

To determine if there is correlation between the protonation state and the solvent exposure of a residue, we monitored the number of water molecules interacting with the residue sidechain. In Figures S14 and S15 we plot the average number of water molecules that are within 0.3 nm (as measured from the water oxygen atom) from the amino acid sidechain, as a function of pH. These hydration values were obtained as averages over the trajectories of all replicas of each pH value. For Asp and Glu, the hydration increases with pH, while for His the hydration decreases with pH. With the exception of Asp48 and Asp66 in HEWL, the hydration curves correlate nicely with the titration curves. These observations suggest that exposure to water has an important effect on the proton affinity of a residue. Because Asp48 and Asp66 are buried within the hydrophobic core of HEWL, access to water is restricted. Instead, we observe that upon deprotonation, these residues form more hydrogen bonds with the protein, as shown in Figure S16.

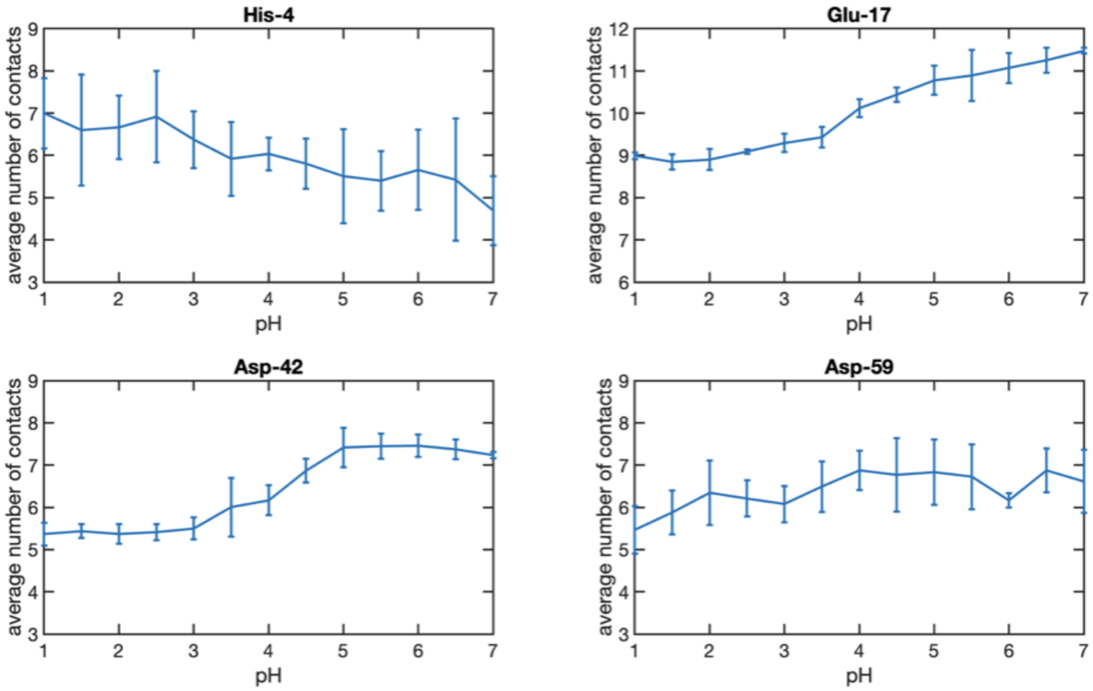

Figure S14: Average number of water molecules within 0.3 nm radius from the residue side chain for cardiotoxin V.

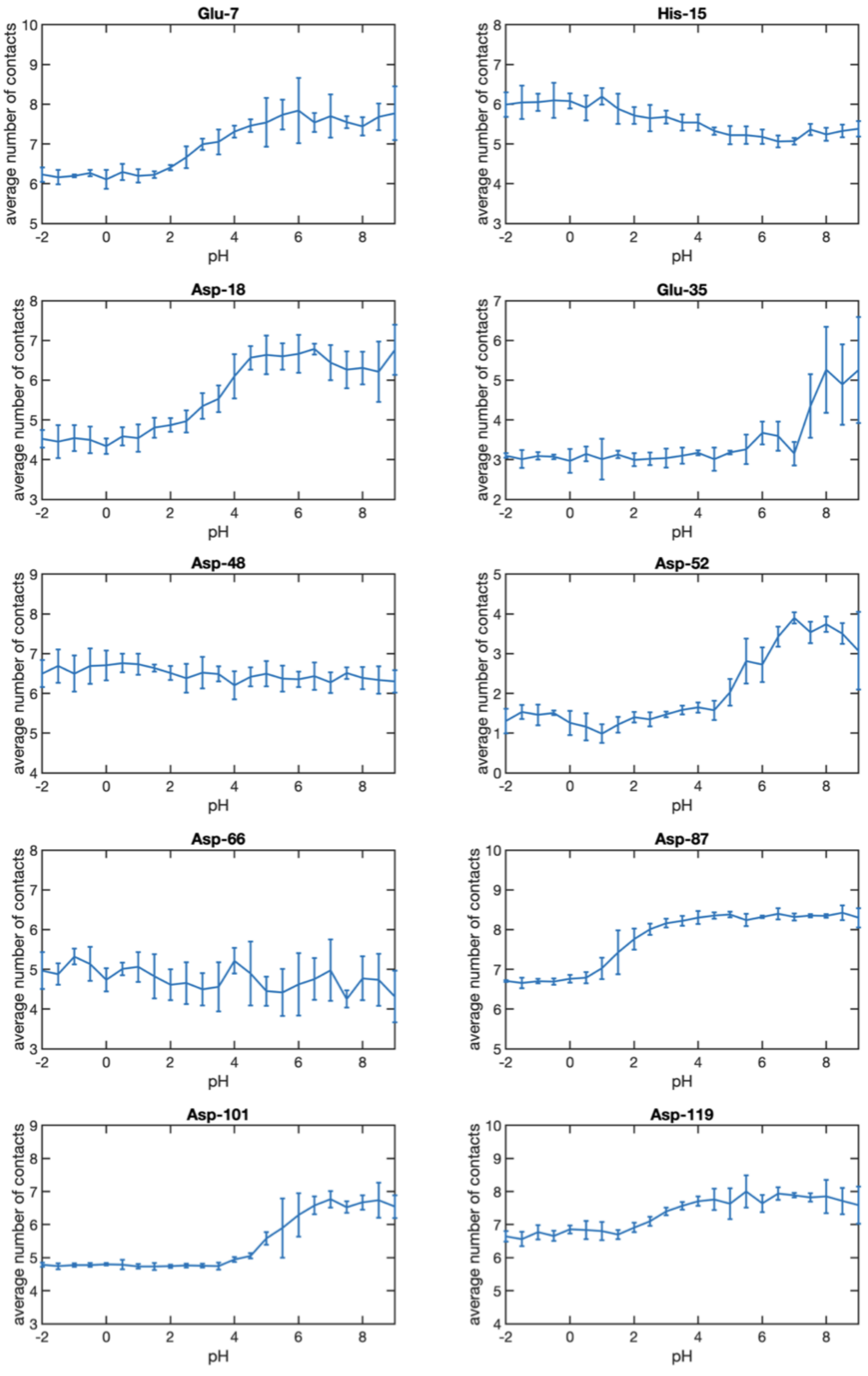

Figure S15: Average number of water molecules within 0.3 nm radius from the residue side chain for HEWL.

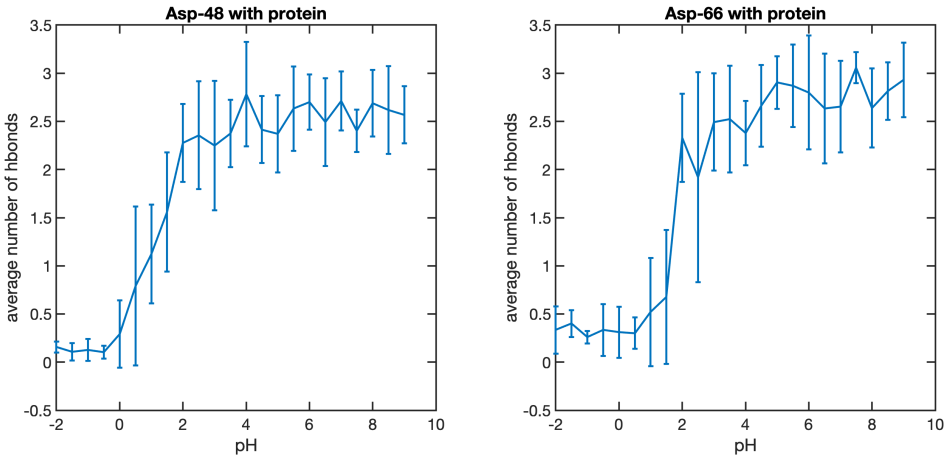

Figure S16: Average number of hydrogen bonds with the rest of protein formed by the side chains of Asp48 and Asp66 of HEWL.

8 Charge interpolation for coupled sites

In the main text, we demonstrated that for "chemically", or topologically uncoupled sites, the gradient of the Coulomb energy with respect to the λ_k coordinates can be evaluated directly from the electrostatic potential if rather than interpolating the Coulomb potential functions linearly, we interpolate the charges linearly instead. Here, we demonstrate that this is also true for chemically coupled sites within the multisite representation.

In the multisite representation, the interpolated charge on an atom i is

$$q_i = \sum_k \left((1-\lambda_k)q_i^0 + \lambda_k q_i^k\right) \tag{S25}$$

where the sum runs over all λ_k-groups that contribute to the charge of that atom. Thus, the total electrostatic interaction of this atom with the other atoms in the system does not depend on the value of a *single*, but on the values of *multiple* λ_k coordinates.

The total λ-dependent electrostatic energy for a system with N_g titratable residues, l, each of which with n_l atoms, and described by ν_l λ_k coordinates coupled within the multisite representation, is:

$$V_{\text{coul}}(\mathbf{R},\boldsymbol{\lambda}) = V^{\text{rest-rest}}(\mathbf{R}) + V^{\mathbf{g}\text{-rest}}(\mathbf{R},\boldsymbol{\lambda}) + V^{\mathbf{g}\text{-}\mathbf{g}}(\mathbf{R},\boldsymbol{\lambda}) + V^{\mathbf{g}}(\mathbf{R},\boldsymbol{\lambda}) \tag{S26}$$

This electrostatic potential has contributions from (i) interactions between atoms that are not part of any titratable residue, $V^{\text{rest-rest}}$; (ii) interactions between the n_l atoms that are part of titratable residue l, and the atoms that are not part of any titratable residue, $V^{\mathbf{g}\text{-rest}}$; (iii) interactions between atoms that belong to different titratable residues, $V^{\mathbf{g}\text{-}\mathbf{g}}$; and (iv) interactions between atoms within the same titratable residue, $V^{\mathbf{g}}$. The first contribution is independent of λ_k, and is not considered further.

Substituting the expression for the total charge on the atoms (Equation S25), in the

second contribution yields:

$$V^{\mathbf{g}\text{-rest}}(\mathbf{R},\boldsymbol{\lambda}) = \sum_l^{N_\mathbf{g}}\sum_i^{n_l}\sum_j^{n_\text{rest}}\sum_k^{\nu_l}\frac{\left((1-\lambda_k)q_i^0+\lambda_k q_i^k\right)q_j}{4\pi\epsilon_0 r_{ij}}$$
$$= \sum_k^{N_{\lambda-\text{groups}}}\sum_i^{n_k}\sum_j^{n_\text{rest}}\frac{\left((1-\lambda_k)q_i^0+\lambda_k q_i^k\right)q_j}{4\pi\epsilon_0 r_{ij}} = V^{\boldsymbol{\lambda}\text{-rest}}(\mathbf{R},\boldsymbol{\lambda}) \quad \text{(S27)}$$

where $N_{\lambda-\text{groups}} = \sum_{l=1}^{N_g}\nu_l$ is the total number of λ-groups in the system, which is equivalent to N_sites used in the main text. Because, in contrast to the single-site representation, in which the λ-coordinate connects two protonation states, each protonation state requires a separate λ-coordinate in the multisite representation, $N_{\lambda-\text{groups}}$ can be larger than N_sites. For example, the histidine side chain has two sites (N_δ and N_ϵ), but is described by three λ-groups. After the substitution, the expression for $V^{\mathbf{g}-\text{rest}}$ is identical to $V^{\boldsymbol{\lambda}-\text{rest}}$ in equation 16 of the main text.

Substituting the expression for the total charge on the atoms (Equation S25) also in the contribution describing the interactions between atoms that are part of different titratable residues, yields:

$$V^{\mathbf{g}\text{-}\mathbf{g}}(\mathbf{R},\boldsymbol{\lambda}) = \sum_l^{N_\mathbf{g}}\sum_{m;m\neq l}^{N_\mathbf{g}}\sum_i^{n_l}\sum_j^{n_m}\sum_k^{\nu_l}\sum_t^{\nu_m}\frac{\left[(1-\lambda_k)q_i^0+\lambda_k q_i^k\right]\left[(1-\lambda_t)q_j^0+\lambda_t q_j^t\right]}{4\pi\epsilon_0 r_{ij}}$$
$$= \sum_k^{N_{\lambda-\text{groups}}}\sum_{t,\mathbf{g}_l(\lambda_k)\neq\mathbf{g}_m(\lambda_t)}^{N_{\lambda-\text{groups}}}\sum_i^{n_k}\sum_j^{n_t}\frac{\left((1-\lambda_k)q_i^0+\lambda_k q_i^k\right)\left((1-\lambda_t)q_j^0+\lambda_t q_j^t\right)}{4\pi\epsilon_0 r_{ij}} \quad \text{(S28)}$$
$$= V^{\boldsymbol{\lambda}\text{-}\boldsymbol{\lambda},\ \text{different groups}}(\mathbf{R},\boldsymbol{\lambda}),$$

with $\mathbf{g}_l(\lambda_k)$ denoting the l-th titratable residue, which has λ_k-coordinate under the multisite constraint. Typically $\mathbf{g}_l(\lambda_k)$ constitutes a residues with ν_l protonation states. Here we have again replaced the combined sums over the $N_\mathbf{g}$ and ν by sums over $N_{\lambda-\text{groups}}$. The superscript "$\boldsymbol{\lambda}$-$\boldsymbol{\lambda}$, different groups" indicates that the Coulomb potential is computed with the interpolated charges of atoms that belong to two different groups, thus not within the same residue.

The last contribution to interaction energy S26 is due to the interactions between the atoms of a residue, $V^{\mathbf{g}}(\mathbf{R}, \boldsymbol{\lambda})$. Substituting equation S25 yields:

$$
\begin{aligned}
V^{\mathbf{g}}(\mathbf{R}, \boldsymbol{\lambda}) &= \frac{1}{2}\sum_l^{N_{\mathbf{g}}}\sum_i^{n_l}\sum_j^{n_l}\frac{\sum_k^{\nu_l}\left[(1-\lambda_k)q_i^0+\lambda_k q_i^k\right]\sum_t^{\nu_l}\left[(1-\lambda_t)q_j^0+\lambda_t q_j^t\right]}{4\pi\epsilon_0 r_{ij}} \\
&= \frac{1}{2}\sum_l^{N_{\mathbf{g}}}\sum_i^{n_l}\sum_j^{n_l}\sum_k^{\nu_l}\frac{\left[(1-\lambda_k)q_i^0+\lambda_k q_i^k\right]\left[(1-\lambda_k)q_j^0+\lambda_k q_j^k\right]}{4\pi\epsilon_0 r_{ij}} \\
&+ \sum_l^{N_{\mathbf{g}}}\sum_i^{n_l}\sum_j^{n_l}\sum_k^{\nu_l}\sum_{t,t\neq k}^{\nu_m}\frac{\left[(1-\lambda_k)q_i^0+\lambda_k q_i^k\right]\left[(1-\lambda_t)q_j^0+\lambda_t q_j^t\right]}{4\pi\epsilon_0 r_{ij}} \\
&= V^{\boldsymbol{\lambda}}(\mathbf{R}, \boldsymbol{\lambda}) + V^{\boldsymbol{\lambda}\text{-}\boldsymbol{\lambda},\text{ same group}}(\mathbf{R}, \boldsymbol{\lambda}),
\end{aligned}
\tag{S29}
$$

Here, the superscript "$\boldsymbol{\lambda}$-$\boldsymbol{\lambda}$, same group" indicates that we compute the Coulomb interactions between atoms belonging to the same group, or residue, using the interpolated charges. Because

$$
V^{\boldsymbol{\lambda}\text{-}\boldsymbol{\lambda},\text{ different groups}}(\mathbf{R}, \boldsymbol{\lambda}) + V^{\boldsymbol{\lambda}\text{-}\boldsymbol{\lambda},\text{ same group}}(\mathbf{R}, \boldsymbol{\lambda}) = V^{\boldsymbol{\lambda}\text{-}\boldsymbol{\lambda}}(\mathbf{R}, \boldsymbol{\lambda})
$$

the final expression for total electrostatic interaction energy when the multisite representation is used to model chemically coupled sites, is the same as for the uncoupled case (Equation 16 in the main text):

$$
\begin{aligned}
V_{\text{coul}}(\mathbf{R}, \boldsymbol{\lambda}) &= V^{\text{rest-rest}}(\mathbf{R}) + V^{\mathbf{g}\text{-rest}}(\mathbf{R}, \boldsymbol{\lambda}) + V^{\mathbf{g}\text{-}\mathbf{g}}(\mathbf{R}, \boldsymbol{\lambda}) + V^{\mathbf{g}}(\mathbf{R}, \boldsymbol{\lambda}) \\
&= V^{\text{rest-rest}}(\mathbf{R}) + V^{\boldsymbol{\lambda}\text{-rest}}(\mathbf{R}, \boldsymbol{\lambda}) + V^{\boldsymbol{\lambda}\text{-}\boldsymbol{\lambda},\text{ different groups}}(\mathbf{R}, \boldsymbol{\lambda}) \\
&+ V^{\boldsymbol{\lambda}\text{-}\boldsymbol{\lambda},\text{ same group}}(\mathbf{R}, \boldsymbol{\lambda}) + V^{\boldsymbol{\lambda}}(\mathbf{R}, \boldsymbol{\lambda}) \\
&= V^{\text{rest-rest}}(\mathbf{R}) + V^{\boldsymbol{\lambda}\text{-rest}}(\mathbf{R}, \boldsymbol{\lambda}) + V^{\boldsymbol{\lambda}\text{-}\boldsymbol{\lambda}}(\mathbf{R}, \boldsymbol{\lambda}) + V^{\boldsymbol{\lambda}}(\mathbf{R}, \boldsymbol{\lambda})
\end{aligned}
\tag{S30}
$$

References

(1) Donnini, S.; Ullmann, R. T.; Groenhof, G.; Grubmüller, H. Charge-neutral constant pH molecular dynamics simulations using a parsimonious proton buffer. *Journal of chemical theory and computation* **2016**, *12*, 1040–1051.

(2) Amemiya, T. *Advanced Econometrics*; Harvard University Press, 1985.

(3) Thurlkill, R. L.; Grimsley, G. R.; Scholtz, J. M.; Pace, C. N. pK values of the ionizable groups of proteins. *Protein science* **2006**, *15*, 1214–1218.

(4) Tanokura, M. 1H-NMR study on the tautomerism of the imidazole ring of histidine residues: I. Microscopic pK values and molar ratios of tautomers in histidine-containing peptides. *Biochimica et Biophysica Acta (BBA)-Protein Structure and Molecular Enzymology* **1983**, *742*, 576–585.

(5) Bürgi, R.; Kollman, P. A.; van Gunsteren, W. F. Simulating proteins at constant pH: An approach combining molecular dynamics and Monte Carlo simulation. *Proteins: Structure, Function, and Bioinformatics* **2002**, *47*, 469–480.

(6) Bennett, C. H. Efficient estimation of free energy differences from Monte Carlo data. *Journal of Computational Physics* **1976**, *22*, 245–268.

(7) Huang, Y.; Chen, W.; Wallace, J. A.; Shen, J. All-atom continuous constant pH molecular dynamics with particle mesh Ewald and titratable water. *Journal of chemical theory and computation* **2016**, *12*, 5411–5421.

(8) Goh, G. B.; Hulbert, B. S.; Zhou, H.; Brooks III, C. L. Constant pH molecular dynamics of proteins in explicit solvent with proton tautomerism. *Proteins: structure, function, and bioinformatics* **2014**, *82*, 1319–1331.

(9) Webb, H.; Tynan-Connolly, B. M.; Lee, G. M.; Farrell, D.; O'Meara, F.; Søndergaard, C. R.; Teilum, K.; Hewage, C.; McIntosh, L. P.; Nielsen, J. E. Remeasuring

HEWL pKa values by NMR spectroscopy: Methods, analysis, accuracy, and implications for theoretical pKa calculations. *Proteins: Structure, Function, and Bioinformatics* **2011**, *79*, 685–702.

(10) Swails, J. M.; Roitberg, A. E. Enhancing conformation and protonation state sampling of hen egg white lysozyme using pH replica exchange molecular dynamics. *Journal of chemical theory and computation* **2012**, *8*, 4393–4404.
